# Preoperative expiratory muscle training for swallowing function in patients with esophageal cancer undergoing esophagectomy: A randomized controlled phase II trial protocol

**DOI:** 10.1371/journal.pone.0344456

**Published:** 2026-03-12

**Authors:** Hiroki Mizusawa, Masaya Noguchi, Tomomi Tamura, Masashi Shiraishi, Kengo Kanki, Osamu Shiraishi, Yoko Hiraki, Hiroaki Kato, Yuji Higashimoto, Takushi Yasuda

**Affiliations:** 1 Department of Rehabilitation Medicine, Kindai University Hospital, Osaka, Japan; 2 Faculty of Medicine, Department of Surgery, Kindai University, Osaka, Japan; 3 Faculty of Medicine, Department of Rehabilitation Medicine, Kindai University, Osaka, Japan; Fujita Health University: Fujita Ika Daigaku, JAPAN

## Abstract

Esophagectomy is a highly invasive and curative procedure for esophageal cancer. Although minimally invasive techniques reduce the incidence of pulmonary complications, postoperative dysphagia remains a common and clinically significant issue. Preoperative expiratory muscle training (EMT) may improve swallowing function by strengthening the relevant muscles; however, its effectiveness in patients with esophageal cancer has not been widely studied. This phase II randomized, controlled, double-blind trial has been designed to evaluate the effects of preoperative EMT on postoperative swallowing function in patients undergoing esophagectomy for thoracic esophageal squamous cell carcinoma. Forty patients will be randomly assigned (1:1) to either the EMT or sham-EMT group. EMT will be performed using the EX-1 Medic^®^ device at 50–70% maximal expiratory pressure, whereas the control group will receive minimal resistance (10 cmH₂O). The primary outcome is the Penetration-Aspiration Scale score on the postoperative video-fluoroscopic swallowing study, targeted at postoperative days 10 (allowable window: postoperative days 6–14). The secondary outcomes will be assessed perioperatively (preoperative and/or postoperative, depending on the measure) and include tongue pressure, the Repetitive Saliva Swallowing Test, T he eating assessment test-10, respiratory muscle strength, appendicular skeletal muscle index, and exercise tolerance. This study has been registered at University Hospital Medical Information Network (UMINID:000057795). Recruitment began on June 10, 2025, is expected to continue until September 30, 2028. This trial will clarify whether preoperative EMT can improve swallowing function and reduce postoperative dysphagia in patients with esophageal cancer, potentially establishing a novel prehabilitation strategy in surgical care. **Trial registration**: UMIN Clinical Trials Registry (UMIN-CTR), UMIN000057795. Registered on May 8, 2025. https://center6.umin.ac.jp/cgi-open-bin/ctr/ctr_view.cgi?recptno=R000065994.

## Introduction

Esophageal cancer remains one of the leading causes of cancer-related mortality worldwide and was the sixth most common cause of cancer-related death in 2017 [1,2]. Esophagectomy remains a cornerstone, potentially curative treatment for localized and locally advanced esophageal cancer [3,4]; however, it is one of the most invasive thoracic procedures and is associated with high rates of postoperative morbidity. Although minimally invasive techniques have reduced postoperative pulmonary complications [5], dysphagia continues to be a common and clinically significant complication after esophagectomy, reflecting the multifactorial impact of surgery on swallowing function. In a 2022 prospective study involving 304 patients with esophageal cancer, the incidence of post-esophagectomy dysphagia was 37% (112 patients), with low body mass index, advanced age, and recurrent nerve palsy identified as independent risk factors [6]. Therefore, even as esophagectomy becomes more minimally invasive, the rate of postoperative dysphagia is unlikely to significantly decrease, as surgical indications will expand to include older patients and those with lower body mass indexes.

Several studies have reported associations between preoperative physical indicators of esophagectomy and postoperative dysphagia. In a study of patients with esophageal cancer who underwent esophagectomy, recurrent nerve palsy (odds ratio [OR]: 6.6, 95% confidence interval [CI]: 1.30–33.8), followed by a smaller midsagittal area of the geniohyoid muscle measured on preoperative computed tomography (OR: 3.6, 95% CI: 1.16–11.1), were significant predictors of postoperative dysphagia [7]. Other studies have reported that perioperative decrease in tongue pressure, as an index of suprahyoid and swallowing muscle strength, predicts the prevalence of aspiration pneumonia after esophagectomy [8]. Another important factor is the association between reduced laryngeal elevation and aspiration observed on postoperative swallowing study after esophagectomy [9]. A more effective and efficient method for training swallowing muscles and function during the limited preoperative period must be established.

Patients undergoing esophagectomy for esophageal cancer represent a clinically relevant population because postoperative dysphagia is frequent and closely associated with aspiration-related pulmonary morbidity. This clinical context also encompasses both patient-related vulnerabilities and procedure-related factors. We suggest expiratory muscle training (EMT) as an intervention to enhance the strength of the swallowing muscles and improve swallowing function in patients with esophageal cancer. EMT involves the application of expiratory resistance during exhalation. Several studies have investigated EMT as an intervention for dysphagia, primarily in patients with stroke [10]. Physiological studies support EMT by demonstrating load-dependent increases in velopharyngeal closing pressure, accompanied by increased palatal and pharyngeal electromyographic activity during expiratory loading [11]. EMT has also been shown to improve orofacial muscle strength and increase suprahyoid muscle activation after training [12]. In a surface electromyography study, Wheeler et al. reported increased activity of the submental muscle complex during expiratory pressure threshold training tasks, indicating that EMT engages swallowing-related submental musculature [13]. These physiological findings provide a rationale for EMT as a candidate approach to target muscles relevant to swallowing. A meta-analysis of randomized controlled trials comparing EMT with control (non-intervention or sham-EMT) in patients with post-stroke dysphagia reported an average reduction of 0.81 points in the Penetration-Aspiration Scale (PAS) score (95% CI: −1.19 to −0.43; P < 0.0001) [10]. These results suggest that EMT may reduce aspiration risk and improve swallowing safety, supporting its clinical utility in dysphagia rehabilitation. In addition, EMT enables standardized, quantifiable load prescription based on a fixed proportion of maximal expiratory pressure, which is feasible within the limited preoperative period. We plan to apply EMT to patients with perioperative esophageal cancer to investigate whether EMT improves swallowing function and mitigates postoperative decline following esophagectomy.

### Objectives

This study aims to investigate, through a randomized controlled trial (EMT vs. sham-EMT) design, whether preoperative EMT can enhance preoperative swallowing function and attenuate postoperative decline in patients with thoracic esophageal cancer scheduled for surgery.

## Methods

### Study design

A prospective, double-blind, randomized controlled trial (phase II) will be conducted at Kindai University Hospital. This study protocol was approved by the Ethics Committee of Kindai University Faculty of Medicine (no. R06-234, approved on April 9, 2025). The study will abide by the ethical standards established in the 1964 Declaration of Helsinki and subsequent amendments. Substantive amendments will be submitted to the institutional review board (IRB)for approval prior to implementation and reflected in the trial registry record. All participants will receive sufficient information about the study, and their written informed consent will be obtained. The trial was registered in UMIN-CTR before enrolment (Registry number: UMIN000057795; registered date: May 8, 2025). The authors confirm that all ongoing and related trials for this intervention are registered, and that any future trials will be registered prospectively. Recruitment began on June 10, 2025, and is expected to continue until September 30, 2028. Follow-up period will be August 1, 2025, to March 30, 2029. Analysis is planned through December 31, 2029. Results will be disseminated through peer-reviewed publications and scientific meetings, and by updates to the UMIN-CTR record. Upon request, a plain-language summary will be provided to participants.

### Participants

The participants are patients with thoracic esophageal squamous cell carcinoma receiving neoadjuvant chemotherapy, followed by esophagectomy at Kindai University Hospital, Osaka, Japan. Key intraoperative and perioperative variables that may influence postoperative dysphagia will be recorded and summarized by group, including the surgical approach and extent of lymphadenectomy, operative time, blood loss, and postoperative recurrent laryngeal nerve palsy. These variables will be compared descriptively between groups to assess balance achieved by randomization.

### Eligibility criteria

1) Patients over 40 years of age
2) Patients undergoing first-line treatment for esophageal cancer
3) Patients with no severe complications in major organs (e.g., bone marrow, heart, liver, kidneys)
4) Patients who can provide written consent after the study is fully explained to them
5) Patients who can walk independently.

### Exclusion criteria at the time point of assessment for eligibility

1) Patients scheduled for two-phase esophageal reconstruction
2) Patients scheduled for laryngectomy
3) Patients receiving nasogastric feeding
4) Patients with contraindications to the expiratory muscle training device (EX-1Medic ^®^)
  - Patients with asthma and frequent exacerbations
  - Patients with ruptured tympanic membrane or other injuries
  - Patients with significant increases in left ventricular end-diastolic volume and pressure
  - Patients with worsening signs or symptoms of heart failure following respiratory muscle training, or those diagnosed by a physician as having a high probability of such worsening
  - Patients with a history of costochondritis dissecans or those diagnosed by a physician as having a high probability of the condition
5) Patients deemed ineligible for the study by the sub-investigator and responsible physician prior to surgery owing to a high risk of pneumothorax from severe emphysematous lesions or giant bullae, which would result in excessive physical invasiveness and be disadvantageous to the patient during expiratory muscle training.

#### Exclusion criteria at the time point of intervention (T1).

Participants who perform EMT/sham-EMT fewer than 4 times per week on average during the intervention period will be considered to have low adherence. These participants will remain included in the full analysis set based on the intention-to-treat principle, while they will be excluded from the per-protocol set defined a priori for sensitivity analyses.

#### Exclusion criteria at the time point of follow-up (T2).

Unable to undergo postoperative follow-up.

- Patients in whom oral intake are unable to initiate within 30 days postoperatively due to surgery-related complications
- Refused to follow-up because of fatigue and/or anorexia

### Randomization and blinding

In this double-blind randomized study, patients will be randomly assigned to either the EMT or sham-EMT group using the nondeterministic minimization method based on three assignment factors, age (<65 or ≥65 years), sex (male or female), and baseline PAS score at enrollment (score 1–2, no dysphagia; 3–5, dysphagia; 6–8, with aspiration). A parallel design will be used in this study. Eligible participants will be assigned to the groups in a 1:1 ratio by an independent individual using the cloud-based case registration allocation system (INDICE cloud) provided by the University Hospital Medical Information Network (UMIN). The intervention type will be disclosed after data collection is completed. Unblinding is permissible only when knowledge of allocation is essential for immediate clinical management. The principal investigator (or designee) will access the assignment via the secure INDICE cloud system and document the rationale, date/time, and personnel involved. The participant will remain in the primary analysis set as randomized.

To maintain blinding, both groups will use the same EMT device; the control group will perform sham-EMT using the identical device. To prevent participants from visually distinguishing resistance settings, the resistance scale/indicator on the device will be covered with colored tape so that the markings are not visible during training. In addition, because the grip area corresponds to the resistance-adjustment component and may be inadvertently manipulated during handling, the adjustment component (handle) will be secured with colored tape to prevent unintended changes in expiratory resistance in both groups. MEP will be measured every 2 weeks. After each MEP assessment, in both groups, the device will be temporarily collected and moved out of the participant’s view; expiratory resistance will be adjusted only in the intervention group based on the prespecified algorithm, whereas the control group will remain at the sham setting. Outcome assessors will remain blinded to group allocation, and participants will be instructed not to disclose any details of their training load or device settings during assessments.

### Interventions

#### EMT group (Intervention).

EX-1 Medic^®^ (POWERbreathe International Ltd., Warwickshire, UK) will be used in the EMT group. Expiratory resistance will be set at 50%–70% of the maximal expiratory pressure (MEP). The intervention in the first week will be set at 50% MEP to allow the participants to become familiar with the equipment and loading. Subsequently, the MEP will be set to 70%. In the three randomized controlled trials on EMT for patients with stroke cited in the meta-analysis, the load pressure of the EMT was fixed at 70% MEP. The MEP was measured every 2 weeks, and the EMT loading pressure was adjusted; accordingly, it ranged between 50% and 70% of the MEP.

#### Sham-EMT group (Control).

EX-1 Medic^®^ will also be used in the sham-EMT group. Expiratory resistance will be set to 10 cmH₂O, the minimum load pressure permitted on the EX-1 Medic^®^. As in the EMT group, MEP will be measured every 2 weeks in the sham-EMT group; however, the load pressure setting will remain unchanged.

### Commonalities between the EMT and sham-EMT groups

Participants in both the EMT and sham-EMT groups will complete at least two sets per day, each consisting of 30 breaths, on a minimum of 4 days per week. However, this protocol will not be followed if adverse effects emerge within 1 week of chemotherapy administration. A daily implementation recording form will be distributed to log the number of trials completed each day.

The preoperative EMT/sham-EMT period is planned to align with the neoadjuvant chemotherapy schedule. Under the standard treatment pathway of two or three chemotherapy cycles, the intended intervention duration is approximately 6–7 weeks. If neoadjuvant chemotherapy is completed after a single cycle, the protocol specifies a minimum intervention duration of 3 weeks. In clinical practice, the preoperative window may be unexpectedly shortened due to changes in chemotherapy or surgical scheduling; in such cases, participants will be encouraged to continue EMT/sham-EMT until the day before surgery whenever feasible. Participants who receive less than 3 weeks of intervention due to an unexpectedly shortened preoperative period will remain included in the full analysis set for the primary intention-to-treat analysis and will be documented as protocol deviations.

The intervention for each group will be conducted preoperatively, and neither group will undergo device-based respiratory training after esophagectomy. All adverse events and serious adverse events will be collected from the point of informed consent collection until the final study visit. Adverse events of special interest include barotrauma, otologic symptoms, presyncope/syncope, bronchospasm, and chemotherapy-related complications during training windows. Severity and relatedness will be assessed by the investigator. Serious adverse events will be reported to the IRB within 24 hours in accordance with institutional policy.

### Outcomes

#### Primary outcome: PAS score during postoperative videofluoroscopic swallowing studies (VFSS).

All VFSS procedures will be performed by certified speech-language pathologists in accordance with standardized radiography protocols. The protocols include the use of appropriately collimated ZEXIRA (Canon Medical Systems, Tokyo, Japan) and Ultimax-i DREX-UI80 (Canon Medical Systems) devices. In accordance with our protocol, participants will be instructed to swallow pre-measured 1 cc and 20 cc boluses of nectar-thick liquid barium sulfate. Each bolus is measured using a graduated medicine cup to maintain volumetric consistency. For the primary endpoint, VFSS will be scheduled for postoperative days 10 (allowable window: postoperative days 6–14), subject to the attending physician’s final clinical judgment regarding the participant’s readiness. The first VFSS performed within postoperative days 6–14 will be used as the primary endpoint assessment. This timeframe was selected to standardize early postoperative VFSS assessment when dysphagia is clinically relevant and VFSS is typically feasible. Similar early postoperative instrumental swallowing assessments after esophagectomy have been performed around postoperative days 7, within postoperative days 7–15, and at approximately two weeks postoperatively [14–16].

The PAS was initially developed and tested by Rosenbek et al. in 1996 to describe aspiration and penetration events [17]. It is an 8-point ordinal scale, where 1 represents the lowest score and 8 represents the most severe score. PAS scores are multidimensional, incorporating several criteria: (1) depth of airway invasion (material above, contacting, or below the level of vocal folds); (2) presence or absence of residual material after swallowing (ejected or not); and (3) the patient’s response to material in the airway (e.g., attempt to clear it) (Table 1). Speech-language pathologists who are blinded to the assignment of the participants will evaluate the VFSS and PAS scores.

**Table 1:** Penetration-Aspiration Scale (PAS) Scoring System and Corresponding Descriptions.

Score	Description of events
**1**	Material does not enter the airway
**2**	Material enters the airway, remains above the vocal folds, and is ejected from the airway
**3**	Material enters the airway, remains above the vocal folds, and is not ejected from the airway
**4**	Material enters the airway, contacts the vocal folds, and is ejected from the airway
**5**	Material enters the airway, contacts the vocal folds, and is not ejected from the airway
**6**	Material enters the airway, passes below the vocal folds, and is ejected into the larynx or out of the airway
**7**	Material enters the airway, passes below the vocal folds, is not ejected from airway despite effort
**8**	Material enters the airway, passes below the vocal folds, and no effort is made to eject

All randomized participants will be included in the primary analysis according to the intention-to-treat principle (full analysis set). Postoperative recurrent laryngeal nerve palsy will not be a criterion for exclusion from the primary analysis. To assess the robustness of the primary findings, we will perform prespecified sensitivity analyses excluding participants with postoperative recurrent laryngeal nerve palsy and exploratory subgroup analyses stratified by the presence or absence of recurrent laryngeal nerve palsy. If recurrent laryngeal nerve palsy results in missing postoperative VFSS within the prespecified assessment period, missing data will be handled using appropriate sensitivity analyses as described above.

#### Other outcomes.

**Fig 1:**
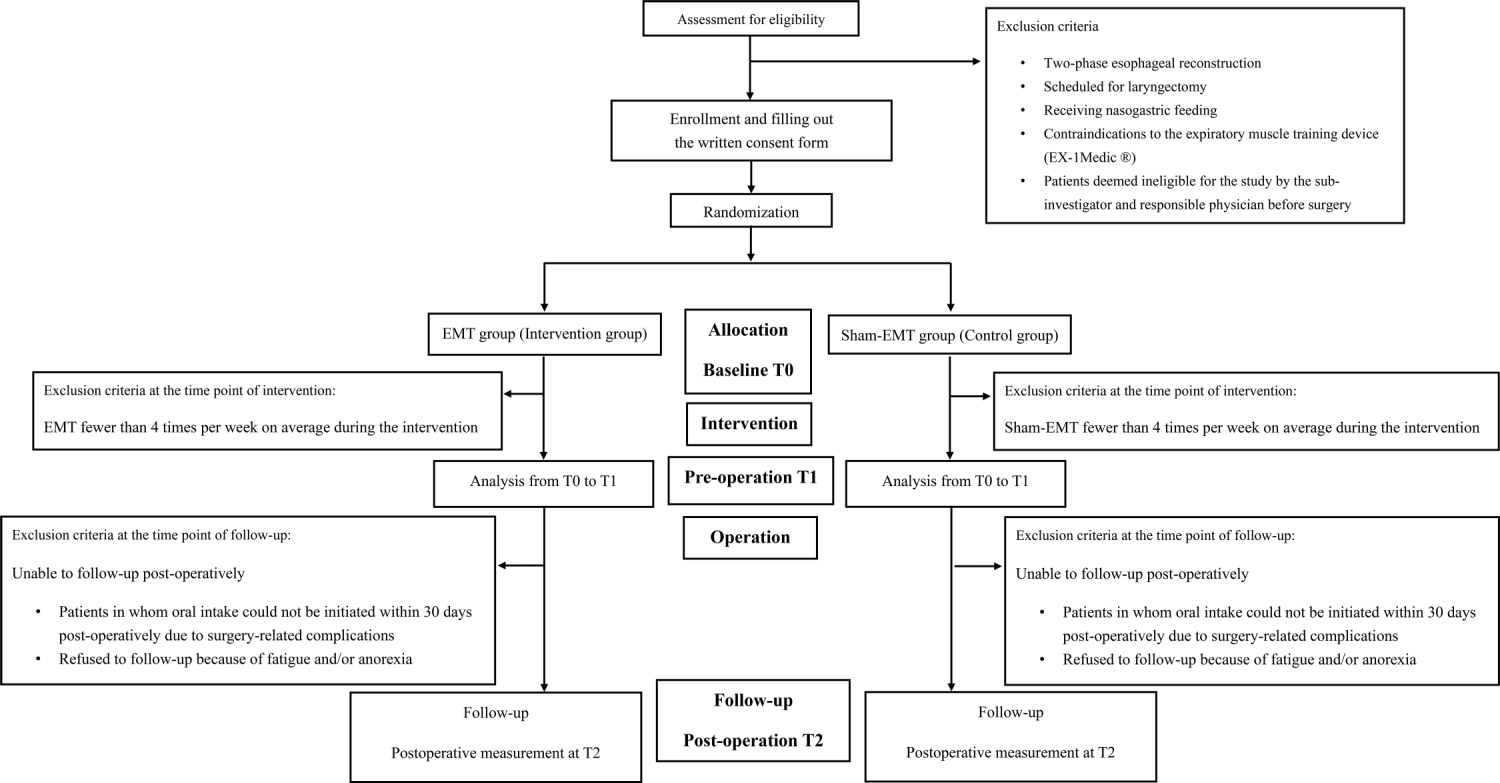
Flow Diagram of Participant Enrollment, Allocation, Follow-up, and Analysis in a Randomized Controlled Trial Evaluating the Effects of Expiratory Muscle Training (EMT) on Postoperative Swallowing Function.

Maximum tongue pressure (MTP) is measured using a balloon-type tongue pressure measurement device (JMS Tongue Pressure Measurement Device® Fig. 1, JMS, Hiroshima, Japan) following the method proposed by Takahashi [18]. The probe is inflated to a reference pressure of 19.6 kPa. When measuring the MTP, the participant places the balloon on the anterior palate with the lips closed, raises the tongue, and presses the balloon against the palate with maximum voluntary force, maintaining this position for approximately 7 seconds. The measurement is performed three times, and the maximum value is recorded as the MTP. The MTP will be evaluated by physical therapists who are blinded to the assignment of the participants. Perioperative tongue pressure reflects oral bolus propulsion and has been linked to swallowing safety, including aspiration risk [8].

For the repetitive saliva swallowing test, the participants will sit in a relaxed posture on a chair and will be instructed to swallow their saliva as many times as possible in 30 s. The examiner will be seated across from the participant, placing the middle finger on the participant’s hyoid and the index finger on the thyroid cartilage. The number of swallows will be counted after confirming that the thyroid cartilage has moved past the middle finger during each swallowing motion. Using a cutoff of ≤2 swallows/30 s on the Repetitive Saliva Swallowing Test, the test identified aspiration/penetration as defined by PAS with 35.5% sensitivity and 92.4% specificity, with performance varying across diagnostic subgroups [19].

The eating assessment test-10 (EAT-10) will be used to assesses patients' self-perception of swallowing function [20]. The EAT-10 consists of 10 items. Each item includes a statement describing a scenario that could be a problem for a subject experiencing swallowing difficulty. Subjects score their symptoms from 0 to 4 (0: no problem, 4: severe problem), with a total score of 3 or higher indicating dysphagia. In a study comparing EAT-10 with VFSS findings, the total EAT-10 score was significantly correlated with PAS and higher scores were shown to identify patients at increased risk of aspiration [21].

A respiratory muscle strength tester (IOP-01; Kobata Keiki Co., Osaka, Japan) will be used to measure the MEP and maximal inspiratory pressure (MIP). The measurements will be repeated until the maximal variation in expiratory or inspiratory effort across the three trials is < 10%, as per the European Respiratory Society statement [22]. The maximum value will be used for the analysis. Based on the report by Hamada et al. [23], predicted MEP values (MEP in men: 25.1 - 0.37 × age + 0.2 × height (cm) + 1.2 × weight (kg); for women: −19.1 - 0.18 × age + 0.43 × height (cm) + 0.56 × weight (kg)) will be used to calculate the percentage of the measured value relative to the predicted value (%pred. MEP). The predicted MIP values (for men: 45 - 0.74 × age + 0.27 × height (cm) + 0.6 × weight (kg); for women: −1.5 - 0.41 × age + 0.48 × height (cm) + 0.12 × weight (kg)) will be used to calculate the percentage of the measured value relative to the predicted value (%pred. MIP).

For the appendicular skeletal muscle index, the measurements will be performed using a Seca mBCA 525 (Seca GmbH & Co. KG, Hamburg, Germany), a portable bioelectrical impedance analyzer. Body weight and height will be measured. The participants will be instructed to remove their socks or stockings and lie supine on the bed. Electrodes will be attached to the dorsum of both hands and feet, and a measurement mat will be placed near the knees and connected to the electrodes. The measurement will then be initiated, and the participants will be instructed to remain still during the procedure, which will last approximately 30 s. The appendicular skeletal muscle index will be calculated by dividing the appendicular skeletal muscle mass by the height (m²), and the resulting values will be used for statistical analysis. Systemic skeletal muscle status was included to characterize sarcopenia, which has been associated with dysphagia in cancer rehabilitation populations and is central to the concept of sarcopenic dysphagia [24,25].

Cardiopulmonary exercise testing on a bicycle ergometer will be conducted in accordance with the guidelines of the American Thoracic Society and American College of Chest Physicians [26]. All patients will undergo the ramp 20-W protocol, which involves a load increase of 20 W per minute, or 2 W every 6 s. Exercise tolerance will be assessed based on peak oxygen consumption (peak VO_2_, mL/min/kg). Exercise tolerance was included as a functional marker of frailty/physiologic reserve; frailty and reduced physical function have been associated with postoperative dysphagia in non-stroke surgical cohorts [27].

### Participant timeline

A flowchart illustrating the study design is shown in Figure 1, and the participant timeline is presented in Table 2. The study will be conducted in accordance with Standard Protocol Items: Recommendations for Interventional Trials. Participants will be assessed for eligibility based on inclusion and exclusion criteria. After providing informed consent, eligible patients will be randomized into either the EMT group (intervention group) or sham-EMT group (control group). Measurements will be performed at baseline (T0), preoperatively (T1), and postoperatively at the time point: immediately after surgery (T2). Reasons for exclusion and dropout at each stage are detailed in the flow diagram (Figure 1).

**Table 2:** Schedule of Enrollment, Intervention, and Assessment for Participants.

Time point	Study period					
	Enrollment	Allocation	Baseline T0	Intervention	Pre-operation T1	Post-operation T2
**Informed consent**	**〇**	**－**	**－**	**－**	**－**	**－**
**Allocation**	**－**	**〇**	**－**	**－**	**－**	**－**
**Videofluoroscopic Swallowing Study**	**〇**	**－**	**－**	**－**	**〇**	**〇**
**Swallowing screening**	**〇**	**－**	**－**	**－**	**〇**	**〇**
**Respiratory muscle strength**	**－**	**－**	**〇**	**〇**	**〇**	**－**
**Tongue pressure**	**－**	**－**	**〇**	**－**	**〇**	**〇**
**Appendicular Skeletal Muscle Index**	**－**	**－**	**〇**	**－**	**〇**	**－**
**Exercise tolerance**	**－**	**－**	**〇**	**－**	**〇**	**－**

### Sample size

Based on the assumption that the EMT intervention improves the PAS score, and referring to the report by Zhang et al., a mean difference in PAS scores of 0.8 was observed between the EMT and sham-EMT groups, with a common standard deviation of 0.8, an alpha error of 0.05, statistical power of 0.8, two-tailed test, and a sample size ratio of 1:1. The total required sample size is 32 (16 cases in a single arm). A total of 40 cases has been set as the minimum target size, accounting for anticipated EMT dropouts (approximately 5% in the previous study) and exclusion of patients with recurrent nerve palsy (approximately 10% of the total).

### Statistical analysis

Statistical analyses will be performed using Statistical Package for the Social Sciences software version 22 (IBM Corp., Armonk, NY, USA). The primary analysis will be conducted in the full analysis set under the intention-to-treat principle, including all randomized participants analyzed as allocated. A per-protocol set will be used for prespecified sensitivity analyses and will exclude participants who do not meet the prespecified adherence criteria. Because recurrent laryngeal nerve palsy can strongly influence swallowing outcomes, we will perform prespecified sensitivity analyses excluding participants with postoperative recurrent laryngeal nerve palsy and exploratory analyses stratified by its presence or absence. If substantial imbalance in major intraoperative factors is observed despite randomization, exploratory covariate-adjusted analyses will be conducted to assess robustness.

Differences in PAS score change scores from baseline to T1 and T2 will be compared between groups using an unpaired t-test, with PAS score analyzed as a continuous outcome. Effect estimates will be reported with 95% CI. Because PAS score is an ordinal scale, a prespecified sensitivity analysis using an ordinal regression approach, such as a proportional-odds model, will be performed. If the proportion of missing values for a primary or key secondary endpoint exceeds 5%, multiple imputations under a missing-at-random assumption will be applied as the main approach, with complete-case analyses presented as sensitivity analyses, and patterns of missingness will be descriptively explored. Secondary outcomes will be interpreted as exploratory, with emphasis on effect sizes and CI. The trial may be paused or modified for safety concerns at the discretion of the principal investigator in consultation with the IRB.

## Limitations

Our primary outcome is based on VFSS-derived PAS score, which primarily evaluates swallowing safety during the oropharyngeal phase. In patients undergoing esophagectomy, dysphagia and aspiration risk may also be influenced by esophageal-phase dysfunction and postoperative reflux or regurgitation resulting from altered anatomy and physiology. These mechanisms may not be fully characterized by our VFSS-based measures and will be acknowledged when interpreting the findings. Where available, routinely documented clinical information such as reflux-related symptoms and relevant postoperative events will be described to provide clinical context. Future studies may incorporate objective assessments of esophageal transit and reflux burden to better delineate their contributions.

This is a single-center study limited to patients with thoracic esophageal squamous cell carcinoma undergoing esophagectomy after neoadjuvant chemotherapy. Accordingly, generalizability to other histology, including adenocarcinoma, and to different treatment settings, such as neoadjuvant chemoradiotherapy, alternative perioperative chemotherapy regimens, or surgery alone, may be limited. To address this, subsequent studies should include broader eligibility across histology and treatment strategies and undertake external validation in multicenter cohorts. In addition, perioperative pathways and rehabilitation practices may vary across institutions; therefore, future multicenter trials should implement harmonized perioperative care and rehabilitation protocols, accompanied by standardized training and quality assurance procedures. Finally, because neoadjuvant chemotherapy regimens may differ over time and between sites, future investigations should adopt prespecified, standardized neoadjuvant regimens or stratify randomization by regimen and center. If efficacy signals are confirmed in this phase II trial, progression to a multicenter phase III trial will be warranted to formally evaluate clinical effectiveness and generalizability.

## Supporting information

S1 Checklist SPIRIT 2025 editable checklist. (DOCX)

S1 File Study_Implementation_Plan_in English. (PDF)

S2 File Study_Implementation_Plan_in_Japanese. (PDF)
