# Supplementary material for: Preoperative expiratory muscle training for swallowing function in patients with esophageal cancer undergoing esophagectomy: A randomized controlled phase II trial protocol
Source: PLoS One. 2026 Mar 12;21(3):e0344456. doi: 10.1371/journal.pone.0344456 (PMC12981427; doi:10.1371/journal.pone.0344456)
Supplement: S1 File — (PDF) [file pone.0344456.s002.pdf]

Phase II Single-blind Randomized Controlled Trial: Effect of Preoperative Expiratory Muscle Training on Postoperative Swallowing Function in Patients with Esophageal Cancer Undergoing Esophagectomy

Study Implementation Plan
Version 1.2

Version 1.2 (Ver. 1.2) — February 12, 2025

Principal Investigator

Yuji Higashimoto, MD, PhD
Department of Rehabilitation Medicine, Faculty of Medicine, Kindai University

Study Office

Department of Rehabilitation, Kindai University Hospital
Hiroki Mizusawa, PT; Masaya Noguchi, PT

Table of Contents

- 1.0 Revision History
- 2.0 Terms and Abbreviations
- 3.0 Study Milestones / Timeline
- 4.0 Background and Rationale
- 5.0 Study Objectives
- 6.0 Methods
- 7.0 Study Initiation, Amendments, and Completion
- 8.0 Statistical Considerations
- 9.0 Scientific Rationale and Study Limitations
- 10.0 Ethical Considerations
- 11.0 Administrative Considerations
- 12.0 Study Organization
- 13.0 References

1.0 Revision History

Version	Date finalized / updated	Summary of changes	Reason for change
1.0	Dec 2, 2024	First version	—
1.1	Jan 7, 2025	Revised following in-person protocol support at KHGRAC	—
1.2	Feb 12, 2025	Revised following in-person data-management support at KHGRAC	—

2.0 Terms and Abbreviations

Term / Abbreviation	English	Definition (Japanese source)
Dean of Faculty of Medicine	—	Dean of the Faculty of Medicine, Kindai University
Ethical Guidelines for Life/Medical Research	—	Ethical Guidelines for Life Science and Medical Research Involving Human Subjects (MEXT/MHLW/METI Notification No. 1, 2021)
Our hospital	—	Kindai University Hospital
Our university	—	Faculty of Medicine, Kindai University
Ethics Committee	—	Ethics Committee, Faculty of Medicine, Kindai University
Declaration of Helsinki	—	World Medical Association Declaration of Helsinki (latest revision)
COI Committee	—	Conflict of Interest Management Committee, Faculty of Medicine, Kindai University

CT	Computed tomography	Computed tomography
EAT-10	Eating Assessment Tool-10	Swallowing screening questionnaire (10 items)
EMT	Expiratory muscle training	Expiratory muscle training
KHGRAC	Kindai Hospital Global Research Alliance Center	—
PAS score	Penetration–Aspiration Scale score	Severity scale for laryngeal penetration/aspiration
PPCs	Postoperative pulmonary complications	Postoperative pulmonary complications
UMIN-CRT	University Hospital Medical Information Network Clinical Trial Registry	Clinical trial registration system operated by UMIN

3.0 Study Milestones / Timeline

Date	Milestone
Dec 2024	Draft Ver. 1.0 completed
Jan 2025	Draft Ver. 1.1 completed
Feb 2025	Draft Ver. 1.2 completed
Apr 2025	Ethics approval; institutional authorization; start patient enrollment
Sep 30, 2028	End of patient enrollment
Mar 31, 2029	End of follow-up
Jun 30, 2029	End of analysis; report study completion to Ethics Committee
Mar 31, 2030	Dissemination of results (manuscript publication)

4.0 Background and Rationale

Esophagectomy with reconstruction is a highly invasive curative treatment for esophageal cancer.[1] Surgery-related complications after esophagectomy include pulmonary complications, anastomotic leakage, anastomotic stricture, recurrent laryngeal nerve palsy, and dysphagia.[2] In our institutional data, the postoperative pneumonia rate decreased from 25.9% (through fiscal year 2019, predominantly open thoracotomy) to 18.0% (fiscal years 2020–2023, after transitioning to minimally invasive approaches), suggesting that postoperative pulmonary complications (PPCs) are declining with less invasive surgery (unpublished data). However, postoperative dysphagia after esophagectomy has remained relatively constant at approximately 15% in our data from fiscal years 2016–2023 (unpublished data).

Prior research in esophageal cancer patients undergoing esophagectomy has reported that recurrent laryngeal nerve palsy (OR 6.6, 95% CI 1.30–33.8) and preoperative CT-measured suprahyoid/geniohyoid muscle cross-sectional area (OR 3.6, 95% CI 1.16–11.1) are significant predictors of postoperative dysphagia.[3] In addition, preoperative tongue pressure, an index of suprahyoid and swallowing-muscle strength, has been reported as a predictor of aspiration pneumonia after esophagectomy.[3] Furthermore, laryngeal elevation is known to significantly influence swallowing after esophagectomy,[2] and reduced laryngeal elevation distance on postoperative videofluoroscopic swallowing studies has been associated with aspiration.[4]

Expiratory muscle training (EMT), which applies expiratory resistance during exhalation, has been investigated as an intervention for dysphagia. Because EMT induces activity in orofacial and pharyngeal muscles (e.g., submandibular, palatal, lingual, and pharyngeal muscles), it may improve muscle strength and cross-sectional area, contribute to the coordination of bolus transport, maintain swallowing safety and efficiency, and lead to functional improvement.[6] A meta-analysis of randomized controlled trials in stroke patients reported that EMT improved the PAS score by −0.81 (95% CI −1.19 to −0.43; I^2=39%; P<0.0001).[6] EMT is believed to enhance muscular and neural adaptations relevant to swallowing recovery, with direct effects in reducing aspiration and pharyngeal residue.[6]

In our group's clinical experience, a male patient in his 50s with COPD who completed four weeks of EMT showed improvements in tongue pressure (48.6→57.7 kPa), Repetitive Saliva Swallowing Test (RSST) (4/30 s→5/30 s), and a swallowing screening questionnaire score. However, all prior randomized trials of EMT for swallowing function have been conducted in stroke patients, and there are no reports of preoperative EMT in patients scheduled for esophagectomy.

We hypothesize that, in patients with esophageal cancer scheduled for surgery, EMT performed during the preoperative neoadjuvant therapy period may attenuate the decline in swallowing function after esophagectomy. This phase II single-blind randomized controlled trial will examine the efficacy of preoperative EMT on postoperative swallowing function and provide foundational data for developing a future multicenter study protocol.

5.0 Study Objectives

(1) To evaluate, in a phase II single-blind randomized controlled trial, the efficacy of EMT performed during the preoperative neoadjuvant therapy period on postoperative swallowing function in patients with thoracoabdominal esophageal cancer scheduled for esophagectomy with reconstruction.
(2) To compare the incidence of aspiration (PAS score ≥6 on postoperative videofluoroscopic swallowing study) between the two groups and estimate the effect size. The estimated effect size will be used to design a future multicenter collaborative study protocol.

Significance: Demonstrating the efficacy of preoperative EMT for preserving postoperative swallowing function in esophageal cancer patients will provide evidence for preoperative rehabilitation interventions aimed at maintaining/improving swallowing function as minimally invasive surgery advances and the surgical population ages.

6.0 Methods

6.1 Study Design

Prospective interventional study; single-blind randomized controlled trial (phase II).

6.2 Study Population

Patients with thoracoabdominal esophageal cancer at Kindai University Hospital who are scheduled to undergo esophagectomy with reconstruction after neoadjuvant therapy.

6.2.1 Inclusion Criteria

- Planned esophagectomy with reconstruction at Kindai University Hospital; scheduled for neoadjuvant therapy; age ≥40 years
- First-line treatment for esophageal cancer
- No severe comorbidity of major organs (bone marrow, heart, liver, kidneys, etc.)
- Written informed consent obtained after adequate explanation
- Independent ambulation

6.2.2 Exclusion Criteria

- Planned two-stage esophageal reconstruction or planned total laryngectomy
- Meets contraindications for EX-1 Medic® EMT device, including: frequent asthma exacerbations; tympanic membrane perforation or similar injury; markedly elevated LV end-diastolic volume/pressure; worsening (or high likelihood) of heart failure signs/symptoms after respiratory muscle training per physician judgment; history or suspected costochondritis
- Active infection or other serious comorbidity such that EMT may increase dyspnea due to respiratory muscle fatigue, reduce consciousness due to hyperventilation, or cause syncope due to rapid hemodynamic changes associated with intrathoracic pressure shifts, as judged by the attending physician
- Difficulty enrolling due to disorientation or psychiatric illness
- High risk of pneumothorax or high physical burden from EMT due to severe emphysematous lesions or giant bullae, judged by investigators prior to surgery
- Serious conditions such as severe pneumonia, heart disease, severe liver disease, severe kidney disease; history of cardiac complications within 1 month (unstable angina, myocardial infarction, percutaneous coronary intervention, coronary artery bypass grafting) or cerebrovascular disease within 6 months; or severe emphysema/giant bullae

6.2.3 Study Devices / Equipment

- Expiratory muscle training: EX-1 Medic® (Entry Japan) (Figure 1)
- Respiratory muscle strength measurement: IOP-01 (Kohata Instrument Manufacturing)
- Exercise capacity: Aeromonitor AE-310S (Minato Medical Science Co., Ltd.)
- Ultrasound for suprahyoid muscles, sternocleidomastoid, and diaphragm thickness: Xario200 (Toshiba)

- Appendicular skeletal muscle mass: seca mBCA 525 (seca)

Note: Use of hospital-managed medical equipment will be approved by the hospital director via the appropriate application process.

6.2.4 Randomization and Allocation

This study is conducted as part of routine preoperative respiratory rehabilitation under health insurance; participants will perform either sham EMT (control) or EMT (intervention) as a component of the rehabilitation program. After obtaining consent, participants will be randomly assigned using the UMIN medical research support (case registration and allocation) system, cloud version [INDICE cloud]. Allocation factors are: age (<65, ≥65), sex (male/female), and baseline PAS score category: 1–2 (no dysphagia), 3–5 (dysphagia), 6–8 (aspiration). Allocation will be performed using a non-deterministic minimization method. These factors are based on reports indicating associations of age and sex with swallowing function, and to balance baseline swallowing function between groups. The INDICE cloud randomization program will be prepared with data-management support from KHGRAC and managed strictly to prevent external disclosure. Participants and outcome assessors will be blinded to allocation.

6.2.5 Interventions

Preoperative and postoperative respiratory rehabilitation programs

Preoperative respiratory rehabilitation (during neoadjuvant therapy period)

Control group (Sham EMT):

- Device instruction and load adjustment during the neoadjuvant therapy period
- Expiratory load fixed at the minimum device setting: 10 cmH_2O
- 30 breaths per set; 2–3 sets/day; completion criterion: ≥5 days/week

Intervention group (EMT):

- Device instruction and load adjustment during the neoadjuvant therapy period
- Load starts at 50% of maximal expiratory pressure and is progressively increased up to 75%
- 30 breaths per set; 2–3 sets/day; completion criterion: ≥5 days/week

Both groups will use a training log to record daily sessions and repetitions.

Postoperative respiratory rehabilitation (during hospitalization)

- Sputum clearance and cough instruction
- Early mobilization
- Range-of-motion exercises for upper and lower extremities
- Strength training (trunk and limbs)
- Whole-body endurance exercise
- EMT will not be performed until 6 months postoperatively.

6.3 Data Source / Database

Clinical records stored in the electronic medical record system.

6.4 Data to be Collected

The following information will be obtained from medical records:

6.4.1 Demographics and Clinical Information

- Age (date of birth), height, weight, body mass index
- Past medical history, comorbidities, allergies (presence and details)
- Diagnosis information (diagnosis and date), clinical stage, clinical outcomes, treatment details
- Medication types

6.4.2 Imaging Examinations

- Videofluoroscopic swallowing study (VFSS)
- Whole-body CT (non-contrast and contrast-enhanced), chest radiography, transthoracic echocardiography
- Results and findings from the above examinations will be used

6.4.3 Pulmonary Function Testing

- Vital capacity, forced vital capacity, FEV_1, diffusing capacity (DLCO)
- Results from the above tests will be used

6.4.4 VFSS

VFSS findings and results obtained at the start of neoadjuvant therapy and postoperatively will be used.

6.5 Endpoints and Assessments

6.5.1 Primary Endpoint

PAS score on videofluoroscopic swallowing study (VFSS).

Rationale

The PAS score obtained from VFSS is considered one of the most accurate and globally used methods for evaluating swallowing function.
PAS score definitions:
1: Material does not enter the airway.
2: Material enters the airway, remains above the vocal folds, and is ejected from the airway.
3: Material enters the airway, remains above the vocal folds, and is not ejected.
4: Material enters the airway, contacts the vocal folds, and is ejected.
5: Material enters the airway, contacts the vocal folds, and is not ejected.
6: Material passes below the vocal folds, and no subglottic residue is visible.
7: Material passes below the vocal folds; patient responds (cough), but residue remains.
8: Material passes below the vocal folds; no patient response; residue remains.
PAS ≥3 is regarded as dysphagia, and PAS ≥6 indicates aspiration.

6.5.2 Secondary Endpoints

- Repetitive Saliva Swallowing Test (RSST)
- Functional Oral Intake Scale (FOIS)
- Maximum tongue pressure
- Ultrasound measures: laryngeal elevation distance; suprahyoid muscle cross-sectional area

Rationale

RSST, tongue pressure measurement, and ultrasound assessment of swallowing and respiratory muscles are non-invasive. RSST is widely used as a swallowing screening measure and can be administered by allied health professionals. Preoperative tongue pressure in esophageal cancer patients has been reported to be associated with the proportion of patients with PAS ≥6 on postoperative VFSS.[5] Preoperative ultrasound-derived laryngeal elevation distance and suprahyoid muscle area have also been reported to relate to postoperative swallowing function. Ultrasound assessment of sternocleidomastoid and diaphragm motion is included to evaluate direct effects of EMT on respiratory muscles. These measures are considered important for interpreting the effect of EMT on swallowing.

6.5.3 Assessment Methods

Respiratory muscle strength

Maximum inspiratory pressure (MIP) and maximum expiratory pressure (MEP) will be measured using the IOP-01 device. MIP is measured by a rapid maximal inspiration from end-expiration; MEP by a rapid maximal expiration from full inspiration. Participants sustain the pressure for ≥2

seconds; the maximum value during the hold is recorded. MIP is measured three times, with confirmation that variability is within 10%; the best value is used. Maximal phonation time is also measured by instructing participants to sustain phonation as long as possible after maximal inspiration. All measures are performed in a seated position.

Swallowing screening tests

Swallowing screening tests include RSST, FOIS, and EAT-10. Assessments will be performed by a co-investigator speech-language-hearing therapist who is blinded to group allocation.

Repetitive Saliva Swallowing Test (RSST)

After moistening the oral cavity, the number of saliva swallows completed in 30 seconds is observed. The assessor palpates the hyoid bone with the index finger and the thyroid cartilage with the middle finger and counts a swallow only when the thyroid cartilage clearly elevates past the palpating finger. Fewer than three swallows in 30 seconds is considered indicative of dysphagia on screening.

Functional Oral Intake Scale (FOIS)

FOIS evaluates oral intake status on a 7-level scale from Level 1 (nothing by mouth) to Level 7 (total oral diet with no restrictions):
Level 1: Tube dependent, no oral intake.
Level 2: Tube dependent with minimal/inconsistent oral intake.
Level 3: Tube supplements with consistent oral intake.
Level 4: Total oral diet of a single consistency.
Level 5: Total oral diet with multiple consistencies requiring special preparation/compensations.
Level 6: Total oral diet with multiple consistencies without special preparation, but with specific limitations.
Level 7: Total oral diet with no restrictions.

Eating Assessment Tool-10 (EAT-10)

EAT-10 is a 10-item questionnaire developed by Belafsky et al. (2008) for dysphagia screening. Each item is rated on a 5-point scale (0=no problem to 4=severe problem). A total score ≥3 suggests swallowing difficulty.

Tongue pressure

Maximum tongue pressure (MTP) will be measured using a balloon-type tongue pressure device (JMS Tongue Pressure Measurement Device®, JMS, Hiroshima, Japan). The balloon is inflated to a baseline pressure of 19.6 kPa. In a seated position, the participant places the balloon against the anterior palate with lips closed, then presses the balloon upward maximally with the tongue for 7 seconds. The test is repeated three times; variability within 10% is confirmed, and the highest value is recorded as MTP.

Ultrasound: laryngeal elevation distance and suprahyoid muscle area

Laryngeal/hyoid elevation distance will be assessed in a seated position using a 3.5 MHz convex probe applied to the submental region with ultrasound gel, avoiding compression of soft tissue. After confirming a mid-sagittal view and the ability to swallow saliva with the probe in place, a resting image is recorded, followed by video recording during spontaneous saliva swallowing. Stored images will be reviewed frame-by-frame (30 frames/s) to identify rest and maximal elevation positions, which are saved as still images. Hyoid displacement distance is then calculated using ImageJ. The displacement is quantified using the rest position (X0,Y0) as origin and maximal elevation (X1,Y1) as the measurement point with cranio-caudal movement as the x-axis and antero-posterior movement as the y-axis. Measurements are repeated three times; the maximum value (with ≤10% variability) is used.

Suprahyoid muscle cross-sectional area will be measured following Macrae et al. A 3.5 MHz convex probe is placed sagittally along the midline of the floor of the mouth at a level that visualizes both the hyoid bone and mandible with acoustic shadowing and the attached geniohyoid muscle. After spontaneous saliva swallowing, still images are saved and the area is calculated by manual tracing in ImageJ.

Appendicular skeletal muscle mass

To verify that whole-body skeletal muscle mass and sarcopenia status do not differ between groups at baseline, appendicular skeletal muscle mass will be measured using seca mBCA 525. After measuring height and weight, participants (without socks/stockings) lie supine on a bed. Electrode pads are placed on the dorsum of both hands and feet and connected to the measurement mat positioned near the knees. Measurement is performed at rest for approximately 30 seconds. The appendicular skeletal muscle index is calculated as appendicular skeletal muscle mass divided by body surface area (m^2) and will be used in statistical analyses.

Exercise capacity

Exercise capacity is associated with PPCs after upper abdominal surgery. It will be assessed to confirm there is no between-group difference at baseline and preoperatively. Either cardiopulmonary exercise testing (CPET) with a cycle ergometer or the 6-minute walk test (6MWT) will be performed.

CPET: Using an expiratory gas analyzer (Minato), a ramp protocol is applied to safely evaluate

exercise capacity, aiming for termination within 8–12 minutes. After 1 minute rest and 1 minute warm-up at 0 W, participants maintain ~50 rpm, then workload increases by 20 W each minute until symptom limitation (dyspnea, leg fatigue) or inability to maintain cadence (<50 rpm). Expected homeostatic changes (e.g., increased breathing and heart rate) should resolve quickly with appropriate rest/hydration.

6MWT: Conducted according to the standard manual. Participants walk as far as possible in 6 minutes at a self-selected pace on a 30 m course (out-and-back) or track. Rest is allowed if needed, and the test may resume after recovery. The total distance walked in 6 minutes is recorded. The test is stopped immediately if SpO_2 falls below 75%.

Assessments performed within routine clinical care

- Respiratory muscle strength measurement
- Swallowing screening tests
- Tongue pressure measurement
- Appendicular skeletal muscle mass measurement
- Exercise capacity assessment

Assessments performed specifically for this study

- Ultrasound assessment of swallowing muscles (laryngeal elevation distance and suprahyoid muscle area)

6.6 Schedule

Preoperative rehabilitation involves instruction, setting, and load adjustment of sham EMT (control) or EMT (intervention). Both groups receive breathing and cough instruction on the day before surgery. Postoperative rehabilitation follows the standard program without EMT; postoperative rehabilitation is routine care as previously practiced, whereas preoperative rehabilitation is performed for this study.

6.6.1 Assessment Schedule

Assessment	Baseline (1–2 days before surgery?)	Start of neoadjuvant therapy	Preoperative	Post-op day 10 ±4	Post-op 1 mo ±7 d	Post-op 3 mo ±7 d	Post-op 6 mo ±7 d
Informed consent	—	●	—	—	—	—	—
Respiratory muscle strength*1	—	●	●	△*2	●	●	●
Swallowing screening*1	—	●	●	△*2	●	●	●
Tongue pressure*1	●	●	●	●	●	●	●
Ultrasound swallowing muscles*1	●	●	●	●	●	●	●

Appendicular skeletal muscle mass	●	●	—	●	●	●	●
Exercise capacity	●	●	—	△*2	△*2	△*2	△*2

*1 Assessments are performed by research staff blinded to participant allocation.

*2 Performed only when approved by the attending physician.

6.6.2 Study Period

Overall study period: From institutional authorization date to March 31, 2030.

Data accumulation / enrollment period: From institutional authorization date to March 31, 2029.

Analysis period: April 1, 2029 to June 30, 2029.

7.0 Study Initiation, Amendments, and Completion

7.1 Initiation

Before starting the study, the Principal Investigator will submit a new application using the designated institutional forms (Kindai Forms 1 and 2), together with the Study Implementation Plan, participant information and consent documents, and consent form. The study will begin after review and approval by the Ethics Committee and authorization by the Dean of the Faculty of Medicine.

7.2 Amendments

If changes or revisions are needed, the Principal Investigator will submit an amendment application using the designated form (Kindai Form 4). Changes will be implemented after Ethics Committee review/approval and institutional authorization by the Dean.

7.3 Completion

Upon completion, discontinuation, or suspension of the study, the Principal Investigator will promptly report to the Dean using the designated form (Kindai Form 6). The study will be discontinued if the Ethics Committee recommends or instructs cessation.

8.0 Statistical Considerations

8.1 Target Sample Size

Control (Sham EMT): 20 participants; EMT: 20 participants; total: 40 participants.

Rationale for sample size

Assuming EMT improves PAS score, and referring to Zhang et al.[6], the mean difference in PAS score between groups is set to 0.8 with a common standard deviation of 0.8. With $\alpha=0.05$ (two-sided), power=0.80, and allocation ratio 1:1, R-based calculation yields 16 participants per group (32 total). Accounting for dropouts (~5% in prior work) and exclusion of patients with postoperative recurrent laryngeal nerve palsy (approximately 10% since 2020), the minimum target sample size is set to 40.

8.2 Statistical Analysis

Patients with postoperative recurrent laryngeal nerve palsy will be excluded from analyses. Between-group comparisons will use t-tests for continuous variables and Fisher's exact test or chi-square tests for categorical variables. Statistical significance will be set at $P<0.05$.

8.3 Definitions

8.3.1 Outcomes

Outcome	Definition	Censoring date
Swallowing function score	PAS score on VFSS (8-point scale)	—
Aspiration incidence	PAS score ≥6 on VFSS	—

8.3.2 Adverse Events

Definitions:
Serious: falls/fractures, cardiopulmonary arrest, death.
Non-serious: hypotension, unsteadiness, dizziness, malaise.
Known: dyspnea, anxiety, palpitations, lower-limb fatigue.
Causality: Adverse events occurring during study procedures (assessment/measurement) are considered related.

Follow-up items when an adverse event occurs: symptoms/findings, onset date, outcome (A=recovered; B=improved; C=unchanged; D=recovered with sequelae; E=death), and outcome date.

Reporting: In the event of a serious or unexpected adverse event, the Principal Investigator will report to the Dean in accordance with institutional regulations. If an adverse event meets criteria for urgent reporting, sub-investigators will immediately notify the Principal Investigator.

Depending on frequency and severity, enrollment may be suspended or the trial interrupted/terminated at the discretion of the study leadership.

9.0 Scientific Rationale and Study Limitations

9.1 Scientific Rationale

If preoperative EMT is shown to attenuate postoperative decline in swallowing function in patients with esophageal cancer, it will provide evidence supporting swallowing-focused interventions as part of preoperative rehabilitation.

9.2 Limitations

- Not double-blinded.
- Short postoperative follow-up period.
- At the time of allocation, the degree of surgical invasiveness for individual patients may not be fully known.

10.0 Ethical Considerations

10.1 Ethical Principles and Guidelines

All investigators will conduct the study in accordance with the Declaration of Helsinki and the Ethical Guidelines for Life Science and Medical Research Involving Human Subjects.

10.2 Informed Consent

Written informed consent will be obtained. Investigators will provide the participant information sheet and consent form, ensuring understandable language, and explain the study both in writing and verbally. After confirming participant understanding, written consent will be obtained via signature. Participants may withdraw consent at any time, and withdrawal requests will be honored.

10.3 Protection of Personal Information

Personal information handling will comply with the Ethical Guidelines, the Act on the Protection of Personal Information, and applicable laws/regulations. Data will be processed so that individuals cannot be identified, and a correspondence table (linkage key) will be created and retained within the university until the end of the retention period. Data will be stored on a computer not connected to the network and recorded to external storage (e.g., USB) kept in a locked cabinet. Study results will be published without identifiable information, and data will not be used for purposes other than this study. Direct identifiers (name, date of birth, etc.) will be removed to prevent re-identification.

The custodian of the correspondence table at Kindai University Hospital will be: Tamotsu Kimura, Department of Rehabilitation, Kindai University Hospital.

10.4 Participant Burden, Risks, and Benefits

The only assessment performed solely for this study is ultrasound measurement of laryngeal elevation distance and suprahyoid muscle cross-sectional area. Other interventions, examinations, and assessments performed during hospitalization (pre/postoperative) are within routine insured medical care and do not incur costs beyond usual patient co-payments. Ultrasound assessments will be conducted with the hospital director's approval for use of hospital-managed equipment, and no costs will be charged to participants.

The EMT device (EX-1 Medic®) will be purchased using research funds of Hiroki Mizusawa and provided to participants. Consumable and equipment-related costs for assessments (see the separate cost calculation document) will be covered by allocated research funds of Professor Yuji Higashimoto. Potential risks include adverse events during assessments; because assessments are conducted during routine clinical hours, physician support from the Department of Rehabilitation Medicine will be available if needed.

Participants will receive the EMT device as a gift; otherwise, there are no direct individual

benefits. However, study findings may contribute to future improvements in care for patients with esophageal cancer.

10.5 Reporting Requirements

The Principal Investigator will report to the Dean using designated forms in the following situations:
(1) Annual progress report on study implementation.
(2) Study completion (or discontinuation/suspension) report.
(3) Deviation report when learning of information/facts that compromise ethical validity or scientific rationale, or could affect study continuation.

11.0 Administrative Considerations

11.1 Data Handling

11.1.1 Data Storage and Disposal

Data will be stored until the later of (a) at least 5 years after study discontinuation/completion, or (b) 3 years after publication of study results.

11.1.2 Secondary Use of Data

This study may involve secondary use of participant data obtained for research purposes. If secondary use is planned, a new research plan will be developed and must be reviewed and approved by the Ethics Committee and authorized by the Dean prior to use.

11.2 Funding and Conflicts of Interest

11.2.1 Funding

Study funding sources: allocated research funds of Professor Yuji Higashimoto (Department of Rehabilitation Medicine, Faculty of Medicine, Kindai University) and research funds of Hiroki Mizusawa.

11.2.2 Conflict of Interest Management

All investigators will disclose conflicts of interest in accordance with institutional policies. The study can be conducted only after review and approval by the COI Committee. Any changes will be reported promptly via an amendment application.

11.3 Public Disclosure

11.3.1 Dissemination of Study Information

After study completion, results will be published in scientific journals and/or presented at academic conferences with appropriate protections for participant privacy. The study will be registered in the UMIN-CRT system and assigned an ID.

11.3.2 Intellectual Property

If economic benefits such as patent rights arise from this study, the rights belong to Kindai University and not to study participants.

11.3.3 Handling of Incidental Findings

Incidental findings that are clinically relevant may be discovered through study assessments. In such cases, the participant will be informed and, if necessary, referred to an appropriate specialist for consultation.

11.4 Monitoring and Audit

This study is considered non-invasive and will not conduct monitoring or audits.

12.0 Study Organization

12.1 Coordinating Institutions (Study Office)

Department of Rehabilitation Medicine, Faculty of Medicine, Kindai University
Department of Surgery, Faculty of Medicine, Kindai University

12.2 Principal Investigator

Yuji Higashimoto, MD, PhD — Clinical Professor, Department of Rehabilitation Medicine, Faculty of Medicine, Kindai University

12.3 Co-investigators

- Hiroki Mizusawa, PT — Department of Rehabilitation, Kindai University Hospital
- Masaya Noguchi, PT — Department of Rehabilitation, Kindai University Hospital
- Tomomi Tamura, ST — Department of Rehabilitation, Kindai University Hospital
- Masashi Shiraishi, PT — Chief, Department of Rehabilitation, Kindai University Hospital
- Osamu Shiraishi, MD — Associate Professor, Department of Surgery, Faculty of Medicine, Kindai University
- Yoko Hiraki, MD — Lecturer, Department of Surgery, Faculty of Medicine, Kindai University
- Hiroaki Katou, MD — Lecturer, Department of Surgery, Faculty of Medicine, Kindai University
- Takushi Yasuda, MD, PhD — Professor and Chair, Department of Surgery, Faculty of Medicine, Kindai University

12.4 Contact Information

Hiroki Mizusawa, PT
Department of Rehabilitation, Kindai University Hospital
Extension (PHS): 8644
Email: hiroki-mizusawa@med.kindai.ac.jp

13.0 References

1. 1. Pennathur A, Gibson MK, Jobe BA, Luketich JD. Oesophageal carcinoma. Lancet. 2013;381(9864):400–412.
2. 2. Low DE, Kuppusamy MK, Alderson D, et al. Benchmarking complications associated with esophagectomy. Ann Surg. 2019;269(2):291–298.
3. 3. Kawata S, Hiramatsu Y, Honke J, et al. Preoperative geniohyoid muscle mass in esophageal cancer patients is associated with swallowing function after esophagectomy. Ann Gastroenterol Surg. 2024;8(6):1026–1035.
4. 4. Vergara J, Andreollo NA, Starmer HM, et al. Swallowing Safety after Remote sub-total Esophagectomy: How Important is Tongue Pressure? Dysphagia. 2024;1–12.
5. 5. Kojima K, Fukushima T, Kurita D, et al. Perioperative decrease in tongue pressure is an intervenable predictor of aspiration after esophagectomy. Dysphagia. 2023;38(4):1147–1155.
6. 6. Zhang W, Pan H, Zong Y, Wang J, Xie Q. Respiratory muscle training reduces respiratory complications and improves swallowing function after stroke: a systematic review and meta-analysis. Arch Phys Med Rehabil. 2022;103(6):1179–1191.
7. 7. Hedström J, Tuomi L, Finizia C, Olsson C. Correlations between patient-reported dysphagia screening and penetration–aspiration scores in head and neck cancer patients post-oncological treatment. Dysphagia. 2018;33:206–215.
8. 8. Sugiya R, Higashimoto Y, Shiraishi M, et al. Decreased tongue strength is related to skeletal muscle mass in COPD patients. Dysphagia. 2022;37(3):636–643.
9. 9. Yanai K, Ito J, Nakahira M, Enoki Y, Sugasawa M. Usefulness of ultrasonography for measuring hyoid movement distance during saliva swallowing: a study in healthy volunteers. Jpn J Head Neck Cancer. 2022;48(4):351–355. (Japanese)
10. 10. Macrae PR, Doeltgen SH, Jones RD, Huckabee ML. Intra- and inter-rater reliability for analysis of hyoid displacement measured with sonography. J Clin Ultrasound. 2012;40(2):74–78.
11. 11. Ogawa N, Ohno T, Kunieda K, Watanabe M, Fujishima I. A novel exercise to improve suprahyoid muscle area and intensity as evaluated by ultrasonography. Dysphagia. 2024;1–9.
