# Supplementary material for: Preoperative expiratory muscle training for swallowing function in patients with esophageal cancer undergoing esophagectomy: A randomized controlled phase II trial protocol
Source: PLoS One. 2026 Mar 12;21(3):e0344456. doi: 10.1371/journal.pone.0344456 (PMC12981427; doi:10.1371/journal.pone.0344456)
Supplement: S2 File — (PDF) [file pone.0344456.s003.pdf]

食道癌患者における術前の呼気筋トレーニングが嚥下機能に及ぼす影響についての **Single-blind randomized controlled trial:**第 2 相試験

研究実施計画書

Ver. 1.2

第 1.2 版（Ver.1.2）　2025 年 2 月 12 日

研究責任者
近畿大学医学部　リハビリテーション医学教室
東本有司

研究事務局
近畿大学病院　リハビリテーション部
水澤裕貴・野口雅矢

目次

1.0　更新履歴 4
2.0　用語・略語 4
3.0　研究の工程 4
4.0　研究の背景・意義 5
5.0　研究の目的 5
6.0　研究の方法 6
6.1　研究デザイン 6
6.2　研究対象 6
6.2.1　選択基準 6
6.2.2　除外基準 6
6.2.3　対象とする医療機器等 7
6.2.4　割付方法 7
6.2.5　介入方法 8
6.3　データベース 8
6.4　観察項目 8
6.4.1　基本情報 8
6.5　評価項目 9
6.5.1　主要評価項目 9
6.5.2　副次評価項目 9
6.5.3　評価項目と評価方法 １０
6.6　スケジュール １４
7.0　研究の開始・変更・終了 １６
7.1　研究の開始 １６
7.2　研究の変更 １６
7.3　研究の終了 １６
8.0　統計的事項 １６
8.1　目標症例数 １６
8.2　統計解析の方法 １７
8.3　定義 １７
8.3.1　アウトカム １７
8.3.2　有害事象 １７
9.0　科学的合理性の根拠及び研究の限界 １８
9.1　本研究の科学的合理性の根拠 １８
9.2　本研究の限界 １８
10.0　倫理的事項 １８
10.1　遵守すべき倫理指針等 １８
10.2　インフォームド・コンセント １８

10.3　個人情報の取扱い……１８
10.4　研究対象者に生じる負担及び予測されるリスク並びに予測される利益……１９
10.5　各種報告……１９
11.0　管理的事項……１９
11.1　情報の取り扱い……１９
11.1.1　情報の保管及び廃棄……１９
11.1.2　情報を将来の研究に用いる可能性について（二次利用）……１９
11.2　研究資金・利益相反……１９
11.2.1　研究資金等……１９
11.2.2　利益相反の管理……２０
11.3　情報公開……２０
11.3.1　研究に関する情報公開……２０
11.3.2　知的財産の帰属……２０
11.3.3　研究により得られた結果等の取扱い……２０
11.4　モニタリング及び監査……２０
12.0　実施体制……２０
12.1　研究代表機関（研究事務局）……２０
12.2　研究責任者……２０
12.3　研究分担者……２０
12.4　本研究に関する連絡先……２１
13.0　参考文献……２１

1.0　更新履歴

Ver.	確定日/更新日	変更内容	変更理由
1.0	2024/12/2	初版	
1.1	2025/01/07	KHGRAC にて対面支援(プロトコル)を受け修正	
1.2	2025/02/12	KHGRAC にて対面支援(DM)を受け修正	

2.0　用語・略語

用語・略語	英語	定義（日本語）
医学部長		近畿大学医学部医学部長
生命・医学系指針		人を対象とする生命科学・医学系研究に関する倫理指針（令和 3 年文部科学省・厚生労働省・経済産業省告示第 1 号）
当院		近畿大学病院
本学		近畿大学医学部
倫理委員会		近畿大学医学部　倫理委員会
ヘルシンキ宣言		世界医師会ヘルシンキ宣言改訂版
COI 委員会		近畿大学医学部利益相反マネジメント委員会
CT	Computed tomography	コンピュータ断層撮影
EAT-10	Eating Assessment Tool-10	嚥下評価ツール-10
EMT	Expiratory muscle training	呼気筋トレーニング
KHGRAC	Kindai Hospital Global Research Alliance Center	―
PAS score	penetration aspiration score	喉頭侵入・誤嚥の重症度スケール
PPCs	postoperative pulmonary complications	術後呼吸器合併症
UMIN-CRT	University Hospital Medical Information Network Clinical Trial Registry	大学病院医療情報ネットワーク研究センター 臨床試験登録システム

3.0　研究の工程

年	工程
2024 年 12 月	Ver.1.0 作成
2025 年 1 月	Ver.1.1 作成
2025 年 2 月	Ver.1.2 作成
2025 年 4 月	倫理委員会承認、研究機関の長の実施許可、患者登録開始
2028 年 9 月 30 日	患者登録終了
2029 年 3 月 31 日	患者フォローアップ終了
2029 年 6 月 30 日	解析終了、倫理委員会へ終了報告
2030 年 3 月 31 日	研究成果の公開（論文化）

4.0　研究の背景・意義

食道癌の根治治療における食道切除再建術は侵襲性の高い治療法である [1]。食道切除再建術後の手術関連合併症には、呼吸器合併症、anastomotic leakage、 吻合部の狭窄、反回神経麻痺、嚥下障害がある [2]。当院のデータより胸部開胸術が中心であった 2019 年度までの術後肺炎の発症率は 25.9%であったのに対し、鏡視下術に切り替わった 2020 年度～2023 年度では 18.0%と減少し、近年の手術低侵襲化により術後呼吸器合併症(Postoperative pulmonary complications ; PPCs)はさらに減少傾向にある(non-published data)。しかしながら、PPCs の減少する一方で食道切除術再建術後の嚥下障害は 2016 年度～2023 年度の当院のデータにおいても一定の割合(約 15%)で推移している(non-published data)。食道切除再建術を行った食道癌患者を対象にした先行研究では、反回神経麻痺(OR:6.6, 95%CI:1.30-33.8)に次いで、術前 CT 画像で測定した顎舌骨上筋の筋断面積(OR:3.6, 95%CI:1.16-11.1)が術後嚥下障害の有意な予測因子とされている [3]。加えて、舌骨上筋や嚥下筋の筋出力指標である術前の舌圧が食道切除再建術後の誤嚥性肺炎の有無の予測因子であることを報告している [3]。食道切除再建術後においては、喉頭挙上運動が食道がん術後の嚥下に有意に影響することが示され [2]、食道切除再建術の術後嚥下造影検査において喉頭挙上距離の減少と誤嚥との関連性を示している [4]。

嚥下障害に対する介入として、呼気時に呼気抵抗を負荷する呼気筋トレーニング(Expiratory muscle training: EMT)を用いた研究報告がある。EMT では、顎下筋、口蓋筋、舌筋、上咽頭筋、咽頭筋などの口腔顔面筋の筋活動を伴うため、筋出力や筋断面積の改善に寄与し、嚥下時のボーラス輸送の調整や嚥下の安全性と効率の維持、および機能的改善に貢献する [6]。主に脳卒中患者に対する EMT の効果について無作為化比較試験を行っている 3 つの論文を用いた、メタアナリシスでは脳卒中患者の EMT の介入において PAS score が 0.81 (95% CI, −1.19 to −0.43; I2=39%; P<.0001)改善することを報告した [6]。 EMT が嚥下機能回復のための筋および神経の適応を改善することが示され、誤嚥や咽頭残留物を減少させる直接的な効果があるとされている [6]。我々の研究グループにおける嚥下に対する EMT 介入例として、当院で 4 週間の EMT を行った 50 代男性の慢性閉塞性肺疾患例では、EMT の介入前後で舌圧が 48.6⇒ 57.7kpa, 反復唾液嚥下テスト 4 回/30 秒⇒5 回/30 秒, 嚥下スクリーニング質問表スコアの改善を認めた。しかし、先行研究の嚥下機能に対する EMT 介入の無作為化比較試験の対象は全て脳卒中患者であり、手術予定の食道癌患者に対する術前 EMT 介入の研究報告はない。我々の仮説では、手術予定の食道癌患者において術前化学療法期間に行う EMT は、食道切除再建術後の嚥下機能低下を抑制する効果が期待できる。今回、第 2 相試験として手術予定の食道癌患者に対する術前 EMT の術後嚥下機能に対する有効性を証明し、今後行う多施設共同研究の研究プロトコル作成の基礎データとする。

5.0　研究の目的

①食道切除再建術予定の胸腹部食道癌患者にする術前補助療法期間中の EMT が術後嚥下機能に及ぼす有効性について、Single-blind randomized controlled trial による第 2 相試験として検証する。②術後嚥下造影検査にて PAS score が 6 点以上の誤嚥発生率を 2 群間で比較し、効果量(effect size)を算出する。算出された effect size を用いて、今後行う多施設共同研究のプロトコルを作成する。

＜意義＞

今回、第 2 相試験として手術予定の食道癌患者に対する術前 EMT の術後嚥下機能に対する有効性を証明し、今後行う多施設共同研究の研究プロトコル作成の基礎データとすることができる。今後、手術の低侵襲化により患者の高齢化が進むなか、手術予定の食道癌患者の嚥下機能の維持・改善における術前リハビリテーション介入のエビデンスの一つとなる。

6.0 研究の方法

6.1 研究デザイン

前向き介入研究, Single-blind randomized controlled trial

6.2 研究対象

近畿大学病院において、術前補助療法後に食道摘出再建術予定の胸腹部食道癌患者

6.2.1 選択基準

対象者は下記のすべての基準を満たし、本人の同意が得られた者とする

1) 近畿大学病院において食道切除再建術を予定されている食道癌患者で、術前補助療法を行う予定の 40 歳以上の患者
2) 食道癌に対して、初回治療の患者
3) 主要臓器（骨髄，心，肝，腎など）に高度な合併症がない患者
4) 本研究の十分な説明が行われた後、患者本人から文書による同意の得られている患者
5) 歩行が自立している患者

6.2.2 除外基準

1) 二期的に食道再建術を実施する予定の患者、喉頭摘出術予定の患者
2) 呼気筋トレーニング機器(EX-1Medic®)の禁忌項目に該当する患者
   ・頻繁に増悪を繰り返す喘息患者
   ・鼓膜破裂などの損傷がある患者
   ・左室拡張末期容積および左室拡張末期圧の著しい上昇がある患者
   ・呼吸筋トレーニング後、心不全の徴候や症状が悪化した患者（もしくは、その可能性が高いと医師により診断された患者）
   ・肋軟骨炎の既往歴がある、またはその可能性が高いと診断された患者
3) 活動性の感染及びその他重篤な合併症[※1]を有し、EMT の実施によって呼吸筋疲労による呼吸困難感の増強、過換気による意識レベルの低下、胸腔内圧の変化に伴う血圧や循環動態の急激な変動による意識消失の有害事象が生じる恐れがあると主治医により判断される場合
4) 見当識障害もしくは精神疾患により，本研究への登録が困難と判断される患者
5) 手術前に試験分担（担当）医師および責任医師が、気胸のリスクが高い高度な気腫化病変や巨大ブラなどにより呼気筋トレーニングの実施に関して患者の身体的な侵襲が高く不利益が生じると判断され，本研究に不適格と判断される患者

※1　重症肺炎，心疾患，重症肝疾患，重症腎疾患など 1 ヶ月以内に心臓合併症（不安定狭心症，心筋梗塞，経皮的冠動脈形成歴，冠動脈バイパス術)、もしくは 6 ヶ月以内に脳血管疾患を患ったことがある患者。また、高度な気腫化病変や巨大ブラがある患者。

6.2.3　対象とする医療機器等

- 呼気筋トレーニング： EX-1 Medic®　(エントリージャパン)　（図 1）
- 呼吸筋力の測定：IOP-01: 木幡計器製作所
- 運動耐容能の測定：エアロモニタ AE-310S (ミナト医科学株式会社)
- 舌骨上筋群、胸鎖乳突筋と横隔膜筋厚の測定：Xario200: THOSHIBA ＊
- 四肢骨格筋量の測定：seca mBCA 525: seca 株式会社

＊病院管理医療機器使用申請書で病院長の承認を得る.

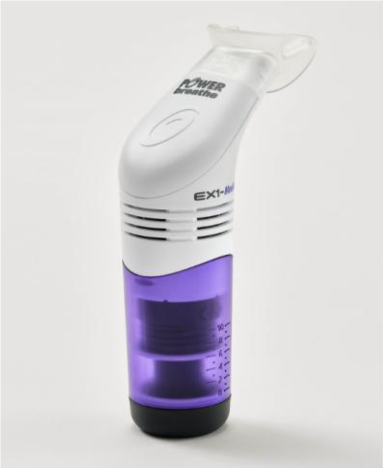

図 1.　EX-1 Medic®　(エントリージャパン)　エントリージャパン HP より

6.2.4　割付方法

本研究は通常保険診療下に実施される術前の呼吸リハビリテーションの一環として実施するが、呼吸リハビリテーションプログラムの一つとして EMT-Sham もしくは EMT を実施する。同意取得後に UMIN 医学研究支援（症例登録割付）システムクラウド版［INDICE cloud］(https://www.umin.ac.jp/indice/cloud.html)にて無作為に群分けを行う。割り付けの際は、年齢(65 歳未満, 65 歳以上)、性別（男性、女性）、Baseline の PAS score : 1-2（嚥下障害なし）, 3-5（嚥下障害あり）, 6-8（誤嚥あり）を割り付け因子として登録し、非決定論的最小化法にて割り付けを行う。割り付け因子の設定には、Hedstrom et al.[7] の報告を参考にし、年齢と性別が嚥下機能に関連することが示されている。さらに、PAS score は造影検査により嚥下機能を評価するツールであり、baseline の時点で EMT-Sham 群と EMT 群の間に嚥下機能に差がないよう割り付け因子に設定する。INDICE cloud における割付プログラムは、KHGRAC のデータマネジメント支援を受けて行う。なお KHGRAC の DM は割付を行うプログラムを外部に漏洩しないように厳重に管理する．また、対象者と評価測定者には割り付け結果を知らせない。

6.2.5　介入方法

術前呼吸リハビリテーションと術後呼吸リハビリテーションプログラム

術前呼吸リハビリテーション（術前補助療法期間）

- **Control 群（Sham-EMT）:**
  術前補助療法期間に行う、機器指導、負荷調整
  呼気負荷は、機器の最低値である 10cmH_2O で常に固定
  30 回 1set, 2~3set/day、最低 5day/week の完遂を包含基準とする

- **介入群 (EMT):**
  術前補助療法期間に行う、機器指導、負荷調整
  最大呼気圧の 50%から漸増，最大 75%まで漸増する
  30 回 1set, 2~3set/day、最低 5day/week の完遂を包含基準とする

両群ともに実施記録表を作成し、日々の実施回数を記録する。

術後呼吸リハビリテーション（入院期間中）

術後呼吸リハビリテーションの内容

Control 群、EMT 群　共通

- 排痰、咳嗽指導
- 早期離床
- 上下肢関節可動域訓練
- 筋力増強訓練（体幹と四肢）
- 全身持久力運動

術後呼吸リハビリテーションにおいて, EMT は術後 6 ヶ月までは実施しない。

6.3　データベース

電子カルテ内に保管された診療記録

6.4　観察項目

本研究では、診療録から以下の情報を取得する。

6.4.1　基本情報

年齢（生年月）、身長、体重、Body mass index

既往歴・併存疾患・アレルギーの有無及び内容

診断情報（診断名と診断日：年月）・臨床病期・臨床転帰・治療内容

服薬の種類

6.4.2　画像検査

嚥下造影検査、全身 CT 画像（単純及び造影）、胸部単純 X 線、心臓超音波画像診断装置 (心臓エコー検査)

上記の検査結果・所見を用いる。

6.4.3　肺機能検査

肺活量(Vital volume capacity)、努力性肺活量(Forced vital volume)、1 秒量、肺拡散能(DLco)

上記の検査結果を用いる。

6.4.4　嚥下造影検査

術前補助療法開始時と術後に行われる嚥下造影検査の結果・所見を用いる。

6.5　評価項目

6.5.1　主要評価項目

嚥下造影検査における PAS score

【設定根拠】

嚥下機能評価として最も精度が高く、世界的に用いられている方法が嚥下造影検査による PAS score の評価方法である。

スコア 1:造影剤が気道に入らず

スコア 2:造影剤が声帯よりも上のレベルで喉頭内侵入するが、喉頭内に残留なし

スコア 3 造影剤が声帯よりも上のレベルで喉頭内侵入し、喉頭内に残る

スコア 4 造影剤が声帯に接するが、嚥下後に喉頭内残留なし

スコア 5: 造影剤が声帯に接し、嚥下後に喉頭内に残る

スコア 6: 造影剤が声門を通過するが、見えるような声門下の残留はなし

スコア 7:造影剤が声門を通過し、患者のむせもあるが、声門下に残留あり

スコア 8: 造影剤が声門を通過し、患者のむせはなく、声門下に残留あり

PAS score が 3 点以上は嚥下障害あり、6 点以上の場合は誤嚥ありと診断される。

6.5.2　副次評価項目

- 反復唾液嚥下テスト(RSST), Functional Oral Intake Scale
- 舌圧
- 超音波画像診断装置による喉頭挙上距離、舌骨上筋の断面積

【設定根拠】

反復唾液嚥下テスト、舌圧測定、および超音波画像診断装置を用いた嚥下筋と呼吸筋の測定は非侵襲的な評価手法である。反復唾液嚥下テストは、嚥下スクリーニング指標として最も使用され、コメディカルでも嚥下機能を評価できるツールである。舌圧測定については、先行研究で食道癌患者の術前の舌

圧値は、食道切除再建術後の嚥下造影における PAS score6 点以上の患者の割合と関連していることを報告している [5]. また、超音波画像診断装置を用いて測定した喉頭挙上距離や舌骨上筋断面積についても、術前の値が食道切除再建術後の嚥下機能に関連していることを報告している。超音波画像診断装置による胸鎖乳突筋と横隔膜移動距離の測定は、EMT における呼吸筋に対する直接的な効果を評価するために測定する。今回の EMT 介入による嚥下機能の影響を考察するうえで、これらの指標は重要であると考えるため測定を行う。

6.5.3　評価項目と評価方法

1. 呼吸筋力測定

   最大吸気圧、最大呼気圧の測定には、 IOP-01(木幡計器)を用いて、最大呼気位から勢いよく素早く最大吸気、もしくは最大吸気位から勢いよく素早く最大呼気を行い測定する（図 2)。その圧を 2 秒以上維持し、その中の最大値を記録する。 最大吸気圧測定を 3 回測定し、誤差 10%以内を確認し、最大値を最大吸気圧(cmH_2O)とする。最長発生時間の測定も併せて行う。最長発生時間は対象者に対して、最大吸気を行った後になるべく長く声を発するように指示し、その発生時間を測定する。上記はいずれも、椅子座位にて行う。

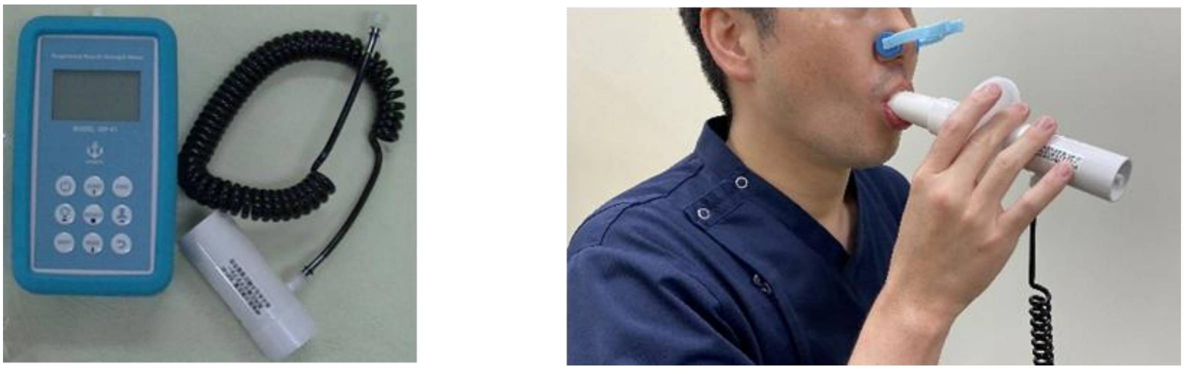

図 2. 呼吸筋力測定器(左図)と実際の測定場面(右図)

2. 嚥下スクリーニングテスト

   嚥下スクリーニングテストは、反復唾液嚥下テスト、Functional Oral Intake Scale、Eating Assessment Tool-10 で評価を行う。いずれも、対象者の割り付けを知らない共同研究者の言語聴覚士により評価測定を行う。

   - 反復唾液嚥下テスト(RSST)

     口を湿らせたのちに 30 秒間に何回唾液を嚥下できるかを観察する。測定者の第２指で舌骨を第３指で甲状軟骨(喉頭隆起)を触知し、甲状軟骨が指を十分に乗り越えた場合のみ１回とカウントする(図 3)。30 秒で 3 回未満は嚥下障害ありとスクリーニングされる。

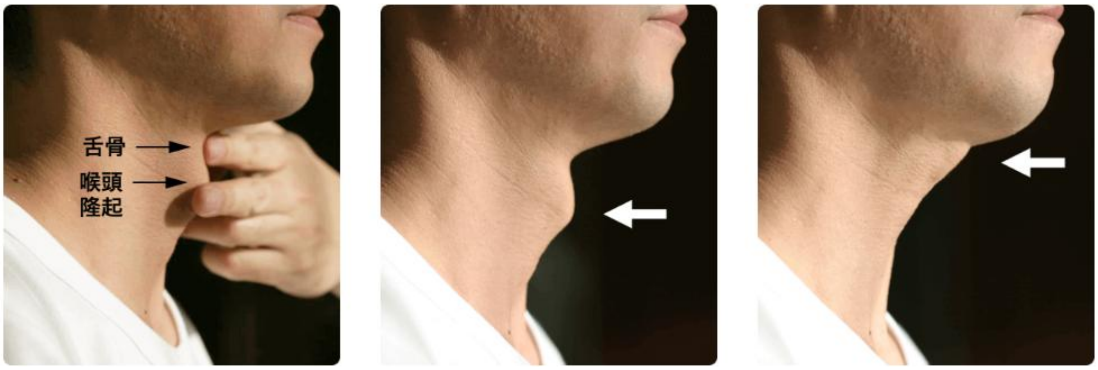

図 3. 実際の測定場面 (出典: https://sapporo-oral-med.jp/swallowing/)

- Functional Oral Intake Scale

  Functional Oral Intake Scale は経口摂取状況についての評価方法で、Level.1「経口摂取なし」から Level.7「正常（制限なく通常の食事ができる状態）」の 7 段階からなる。

  Level.1：経管栄養摂取のみで経口摂取なし

  Level.2：経管栄養中心だが、お楽しみ程度に食物や液体を摂取

  Level.3：経管栄養と経口摂取の併用

  Level.4：一物性のみの経口栄養摂取（ペースト食）

  Level.5：特別な準備もしくは代償法を必要とする複数の物性を含んだ経口栄養摂取（とろみ刻み食）

  Level.6：特別な準備なしだが特定の制限を必要とする複数の物性を含んだ経口栄養（全粥軟菜食）

  Level.7：とくに制限のない経口栄養摂取（常食）

- Eating Assessment Tool-10

  Eating Assessment Tool-10 (EAT-10)は、摂食嚥下障害のスクリーニング評価として 2008 年に Belafsky らにより開発された質問票の評価ツールである。10 項目の質問で構成され、それぞれ 5 段階(0 点 : 問題なし～4 点 : ひどく問題)で回答し、10 項目の合計が 3 点以上となれば摂食嚥下に問題がありと判断される。

3. 舌圧測定

最大舌圧（MTP）は、バルーンを用いた舌圧測定装置（JMS 舌圧測定装置®、JMS、広島、日本）(図 4)を用いて測定した。バルーンはベースライン圧 19.6 キロパスカル（kPa）で膨らます。対象者は、椅子座位姿勢の状態で、唇を閉じた状態でバルーンを前口蓋に装着する。対象者は舌でバルーンを押しつぶすように前口蓋の方向に最大限挙上させ、最大随意筋力でバルーンを 7 秒間押し付ける[8]。これを 3 回繰り返し、誤差 10%以内を確認し、最大値を MTP として記録する。

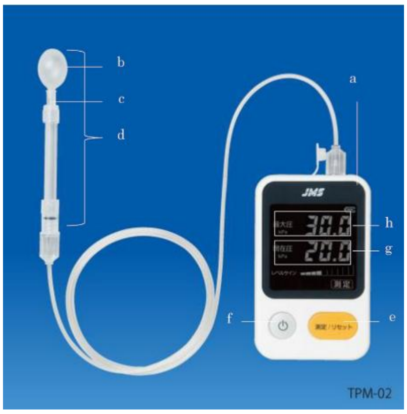

図 4. 実際の舌圧測定器具 (Sugiya et al, Dysphagia 2022)[8]

4. 超音波画像診断装置による喉頭挙上距離、舌骨上筋断面積の測定

超音波画像診断装置による喉頭挙上距離の測定

対象者を頭部背もたれ付きのリクライニング車いすで座位とし、3.5MHz のコンベックス型プローブを顎下部に当てる。その際、超音波ジェルを使用し、オトガイ下の軟部組織を圧迫しないように配慮する。正中矢状断が描出できることを確認した後に、プローブを当てたまま唾液嚥下が可能であることを確認する。舌骨の安静時の記録を行い、静止画として保存する。その後、唾液の自由嚥下を行い、動画として保存する。保存した超音波画像を Windows video player にて 30/秒コマ送り再生にて 1 コマ毎のずれを追跡後、安静位、最大挙上位を確認し静止画として保存する。静止画として保存した画像を image J の Measure (National Institutes of Health, USA)で舌骨移動距離を計算する。なお、舌骨の可動範囲は矢内ら[9]の方法を参考に、頭尾方向移動距離を X 軸、前後方向移動距離を Y 軸とした。安静時の舌骨と舌の付着部分(X0, Y0)を基点とし、唾液嚥下時の舌骨最大挙上位(X1, Y1)を計測点として、舌骨移動距離を測定し、3 回測定し、誤差 10%以内を確認し最大値を解析に使用する(図 5)。

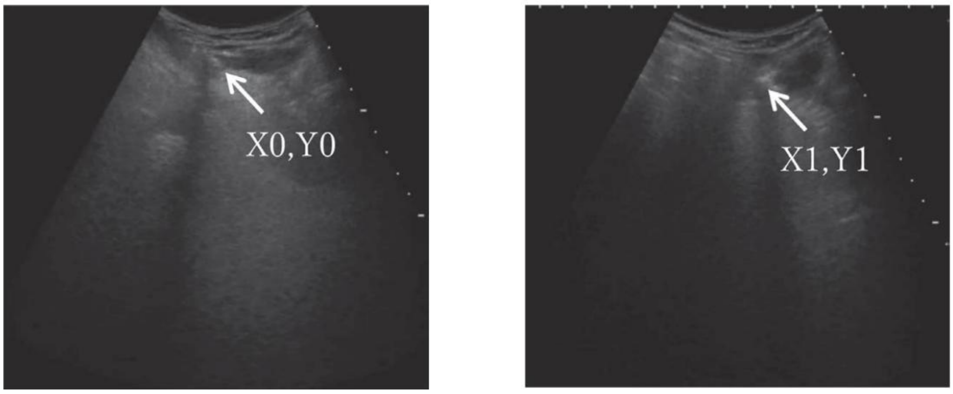

図 5. 左図：安静位、右図：最大挙上位. (矢内ら 頭頚部癌 2022)[8]

超音波画像診断装置による舌骨上筋断面積の測定

Macrae ら[10]の方法を参考に測定を実施する。対象者を頭部背もたれ付きのリクライニング車いすで座位とし、3.5MHz のコンベックス型プローブを矢状面の口底正中線の表面に置き、末

端が甲状軟骨に接しない高さで、かつ舌骨が描出できる高さとする。また、超音波ジェルを十分に使用し、オトガイ下の軟部組織を圧迫しないよう注意する。B モード矢状断層面で、音響陰影を伴った舌骨と下顎骨、それに付着するオトガイ舌骨筋を一つの画面に描出する。その後、唾液の自由嚥下を行い、静止画として保存する。静止画として保存した画像を image J の Measure (National Institutes of Health, USA)でマニュアルトレースにて断面積計算する。

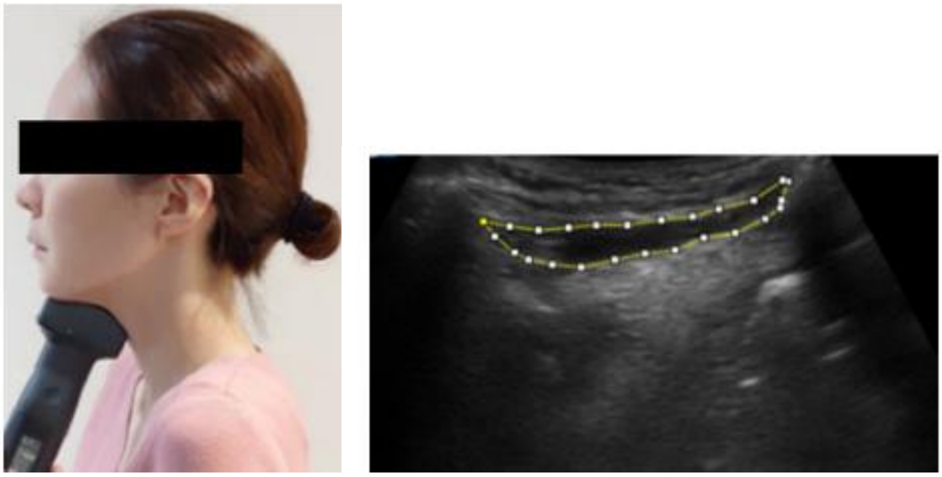

図 6. 実際の測定場面(左図)と測定画面(右図)　(Ogawa et al, Dysphagia 2024)[11]

5.　四肢骨格筋量測定

Baseline での Control 群と介入群で全身の骨格筋量及びサルコペニアの有無に差がないかどうかを検証するために四肢骨格筋量の測定を行う。測定には、seca mBCA 525 を使用する。はじめに体重、身長を測定する。手順は、靴下やストッキングを脱ぎ、ベッド上で仰臥位になり、電極パッドを左右の手背、足背に貼付し、測定マットを膝上付近に設置して電極パッドと接続する。その後、測定を開始する。測定中は安静を保持して頂く（30 秒）。四肢骨格筋指数は、四肢骨格筋量を体表面積(m^2)で除算して算出し、統計解析に用いる。

6.　運動耐容能の測定

上腹部手術において運動耐容能は術後呼吸器合併症に関連する項目であるが、Baseline と術前において Control 群と介入群で運動耐容能について 2 群で差がないかどうかを確認するために測定を行う。運動耐容能の評価は、心肺運動負荷試験もしくは 6 分間歩行試験を行う。

・心肺運動負荷試験

自転車エルゴメーターを用いた心肺運動負荷試験を行い評価する。呼気ガス分析装置（ミナト社製）にて、漸増運動負荷試験を実施する。Lamp 負荷法を用いて実施する。Lamp 負荷法は継時的に負荷量が増大していくため、安全に運動耐容能の評価が可能である。この負荷方法は、正確な能力を評価するために対象者の疲労や運動器への影響を考慮して、8～12 分で終了できるものが望ましいとされており、その時間内に終了する様に負荷量を設定する。運動負荷試験のプロトコールは、安静 1 分間後、0Watt の warm up を 1 分間行い、回転数は 50 回転を目標と

する。その後、1 分間 20W ずつの ramp 負荷を症候限界（呼吸困難、下肢疲労）もしくは回転数（50 回転以下）が維持できなくなるまでを測定する。尚、負荷により生じる身体的な恒常性の変化（呼吸や心拍数の増加等）が適切な休息や補水等により短時間で緩解する。

・6 分間歩行試験
6 分間歩行試験のマニュアルに従って行う。自己のペースで 6 分間に歩くことができる最大距離を測定する検査であり、その距離により運動能力を評価する。片道 30m ある歩行路にて、直線歩行の往復、またはトラックで測定を行う。試験継続が困難な場合は、途中で休憩が可能で、回復した後に再度歩行を行うことができる。6 分間の最大歩行距離を 6 分間歩行距離とする. SpO2 値が<75%を下回った場合には直ちに 6 分間歩行試験を中止する。

通常診療の範囲内で行う検査

- 呼吸筋力測定
- 嚥下スクリーニング検査
- 舌圧測定
- 四肢骨格筋量測定
- 運動耐容能の測定

研究のために行う検査

- 超音波画像診断装置による喉頭挙上距離、舌骨上筋断面積の測定

6.6　スケジュール

6.6.1　スケジュール

術前呼吸リハビリテーションに関しては、Control 群は Sham-EMT、介入群は EMT の指導、設定、負荷調整であり、両群ともに術前日に呼吸指導, 咳嗽指導を行う。術後呼吸リハビリテーションでは、EMT は行わず、通常の術後呼吸リハビリテーションを実施する。術前の呼吸リハビリテーションは、本研究のために実施される。術後の呼吸リハビリテーションは通常診療の範囲内であり、これまでと同様の内容であり通常行うリハビリテーションである。

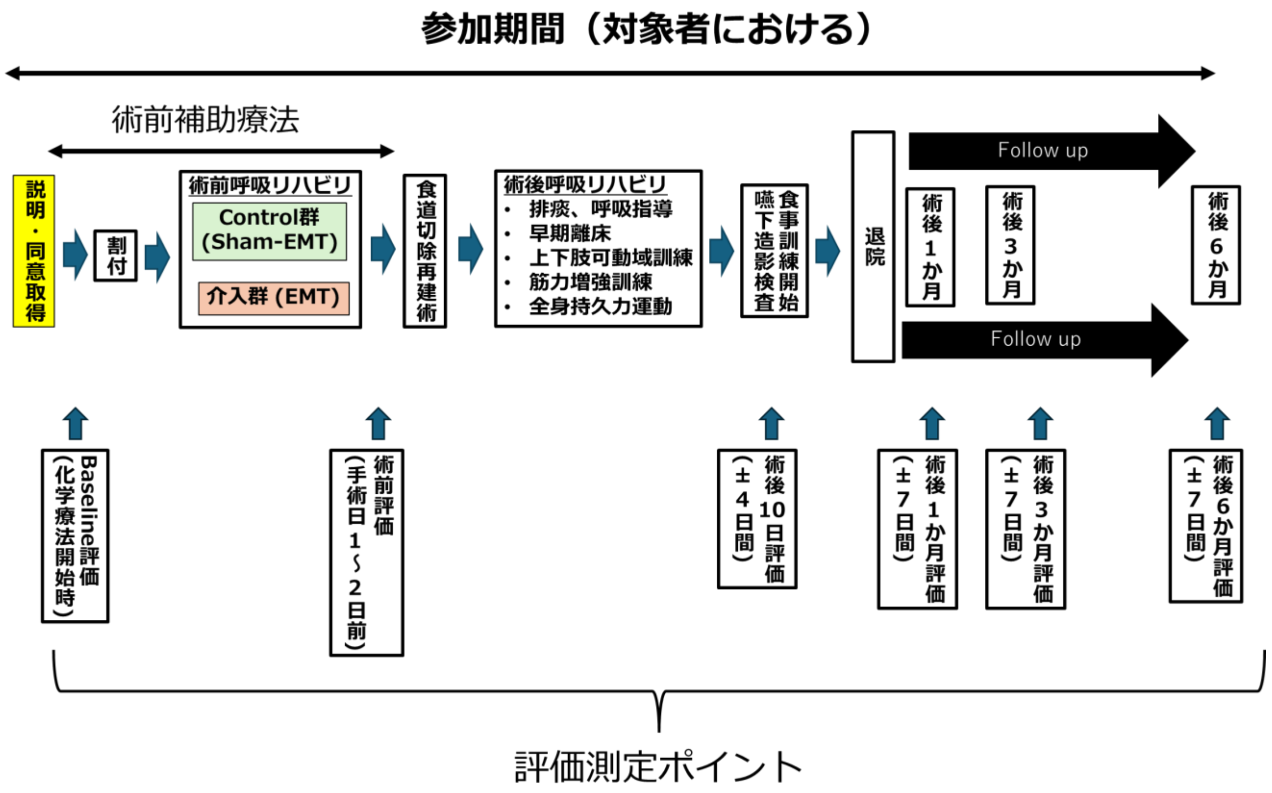

評価項目	Baseline<br>術前補助療法開始時	術前<br>手術日<br>1～2 日前	術後 10 日<br>±4 日間	術後 1 か月<br>±7 日間	術後 3 か月<br>±7 日間	術後 6 か月<br>±7 日間
説明・同意取得	●	―	―	―	―	―
呼吸筋力測定*1	●	●	△*2	●	●	●
嚥下スクリーニング*1	●	●	△*2	●	●	●
舌圧測定*1	●	●	●	●	●	●
超音波画像診断装置による嚥下筋評価*1	●	●	●	●	●	●
四肢骨格筋量測定	●	●	―	●	●	●
運動耐容能の測定	●	●	―	△*2	△*2	△*2

＊1　上記測定は、研究分担者が行うが、対象者の割付を知らされていない者が行う。

＊2　主治医の許可がある場合に行う。

通常診療内(呼吸リハビリテーション)で実施する項目

・呼吸筋力測定

・嚥下スクリーニングテスト

・舌圧測定

・四肢骨格筋量測定
・運動耐容能の測定

本研究用に実施する項目
・超音波画像診断装置による嚥下筋評価

6.6.2　研究期間

研究実施予定期間（全体）	研究機関の長の許可日　～　2030 年 3 月 31 日
データ集積期間 / 症例登録期間	研究機関の長の許可日　～　2029 年 3 月 31 日
解析期間	2029 年 4 月 1 日　～　2029 年 6 月 30 日

7.0　研究の開始・変更・終了

7.1　研究の開始

研究責任者は、本研究を開始する前に所定の様式（【近大-様式 1】及び【近大-様式 2】）、研究実施計画書、同意説明文書、同意書等にて新規申請を行う。倫理委員会における審査を受け、承認された後、医学部長による実施の許可を得る。

7.2　研究の変更

研究責任者は、本研究の変更又は改訂を行う場合、所定の様式 (【近大-様式 4】) にて変更申請を行う。倫理委員会における審査を受け、承認された後、医学部長による実施の許可を得る。

7.3　研究の終了

研究責任者は、本研究の終了時並びに中止又は中断を決定した際には、所定の様式（【近大-様式 6】）にて速やかに医学部長への報告を行う。また、研究責任者は、倫理委員会より本研究に関する中止の勧告又は指示があった場合、本研究を中止する。

8.0　統計的事項

8.1　目標症例数

Control 群(Sham-EMT) 20 例、EMT 群 20 例、合計 40 例

【設 定 根 拠】

EMT 介入により PAS score が改善すると仮定し、Zhang et al.[6] の報告を参考に Control(Sham-EMT)群と EMT 介入群の PAS score の平均値の差を 0.8, 共通する標準偏差を 0.8、αエラー0.05、 検出力 0.8、両側検定、サンプルサイズの比を 1:1 として R を用いて計算すると、片群 16 例、合計 32 例必要と算出された。EMT 脱落例(前回研究時は約 5%)と解析除外となる反回神経麻痺の患者 (8.2 統計解析の方法を参照、2020 年度以降は全体の約 10%）を考慮して、合計 40 例を最低目標症例数として設定した。

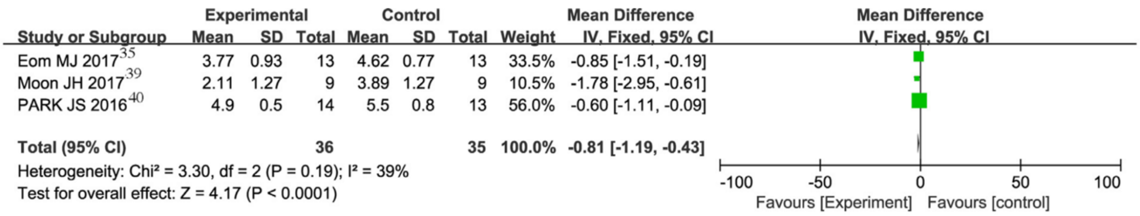

Fig 5 PAS forest plot showing the mean difference (95% CI) of the effect of respiratory muscle training versus no or sham respiratory intervention on PAS score (n=71).

8.2　統計解析の方法

術後に反回神経麻痺を呈した患者は解析除外とする。Control 群と EMT 介入群の 2 群間において、連続変数に関しては t 検定、カテゴリー変数については Fisher の正確検定もしくは χ^2 検定を実施する。有意水準は 5%未満とする。

8.3　定義

8.3.1　アウトカム

アウトカム	定義	打ち切り日
嚥下機能スコア	嚥下造影検査における PAS score により 8 段階で判定する	-
誤嚥発生率	嚥下造影検査における PAS score ≥6	-

8.3.2　有害事象

- 有害事象の定義（重篤・非重篤、既知・未知、因果関係の定義を含む）
  重篤：転倒・骨折、心肺停止、死亡
  非重篤：血圧低下、ふらつき、めまい、気分不良
  既知：呼吸困難感、不安、動悸、下肢疲労感
  因果関係：研究実施時間内（検査測定時）における上記の有害事象は因果関係あり

- 有害事象が発生した場合の追跡項目
  症状・所見、発現日
  転帰（＊転帰　A：回復　B：軽快　C：不変　D：回復したが後遺症あり　E：死亡）
  転帰日

- 有害事象が発生した場合の報告方法
  重篤な有害事象、又は予期されない有害事象が生じた場合、研究責任者は規定に則り医学部長へ報告する。又、緊急速報の対象となる有害事象が発生した場合、研究分担医師は研究責任者へ伝える。発生頻度に応じ研究代表責任医師の判断により登録の中止あるいは試験を中断又は中止する。

9.0 科学的合理性の根拠及び研究の限界

9.1 本研究の科学的合理性の根拠

本研究において、食道癌患者における EMT 介入が術後嚥下機能の低下の抑制作用が明らかになれば、術前リハビリテーションにおける嚥下に対する介入手段のエビデンスとなる。

9.2 本研究の限界

本研究には、以下の限界がある。

・二重盲検ではないこと。

・術後のフォローアップが短期間であること。

・割付の時点では、対象患者の手術の侵襲の程度が把握できないこと。

10.0 倫理的事項

10.1 遵守すべき倫理指針等

本研究に参加するすべての研究者は、ヘルシンキ宣言及び生命・医学系指針に従い、研究を実施する。

10.2 インフォームド・コンセント

本研究に参加するすべての研究者は、以下の方法により、研究対象者の研究参加への同意を取得する。

- 文書による説明と文書による同意

  本研究の研究者は、研究対象者が理解しやすい表現に配慮した説明同意文書･同意書を研究対象者本人に渡した上で、文書及び口頭による十分な説明を行う。研究対象者が研究の内容をよく理解したことを確認した上で、研究対象者本人が研究参加に同意した場合、本研究の同意書を用いて研究対象者本人による署名を得る。研究参加の同意を得た後でも、研究対象者本人から研究参加への同意を撤回する申し出があった場合、同意撤回に応じる。

10.3 個人情報の取扱い

本研究に参加するすべての研究者は、本研究における個人情報の取扱いに関して、生命･医学系指針、個人情報の保護に関する法律及び適用される法令、条例等を遵守する。

本研究で取り扱う情報等は、特定の個人を識別できないように加工した上で、研究・解析に使用する。対照表を作成し、個人情報保管期間が終了するまで本学内に保管する。保管については、ネットワークから切り離されたコンピュータを使用して、USB 等の外部記憶媒体に記録され、ロッカーに鍵をかけて厳重に保管する。本研究の成果を公表する場合にも、研究対象者個人を特定できる情報を含まない形で行い、本研究の目的以外に、本研究で得られた情報を利用しない。加工の方法として、情報から個人を識別できる情報（氏名、生年月日等）を削除し、個人情報を復元できないようにする。

本学における対照表の管理者を、下記のとおり設定する。

本学における対照表の管理者：近畿大学病院　リハビリテーション部　木村保

10.4　研究対象者に生じる負担及び予測されるリスク並びに予測される利益

本研究のみのために行われる検査は、超音波画像診断装置による喉頭挙上距離、舌骨上筋断面積舌骨上筋群の測定である。その他、入院期間中（術前・術後）に本研究で行われる治療及び診察や検査は医療保険の範囲内の行為であり、通常の医療保険制度に沿った対象者の自己負担以外は発生しない。尚、超音波画像診断装置による喉頭挙上距離、舌骨上筋断面積舌骨上筋群の測定についても、病院長に病院管理医療機器使用許可申請書について承諾得て実施し、対象者に検査測定の費用は請求しないため自己負担はない。研究に使用する呼気筋力トレーニング機器(EX-1 Medic®)については、近畿大学病院リハビリテーション部　水澤裕貴の科研費から購入し対象者に配布する。また、検査実施における消耗品等の使用機器の費用(別紙：使用機器の費用計算を参照)については、リハビリテーション医学　東本有司教授の配分研究費から支出する。予測されるリスクとしては、評価測定中の有害事象であるが、通常診療時間に行うため有害事象が生じた場合はリハビリテーション科医師の対応を依頼する。本研究における対象者の利益は、呼気筋トレーニング機器は贈呈となるが、それ以外の直接的な利益はない。しかしながら、今回の研究成果により食道癌患者における将来の医療の進歩に貢献できる可能性がある。

10.5　各種報告

研究責任者は、以下 1～3 の場合に、所定の様式を用いて、医学部長に報告を行う。

1. 研究の実施状況（進捗）に関する定期報告（年 1 回）
2. 研究終了（中止・中断）報告
3. 研究の倫理的妥当性若しくは科学的合理性を損なう事実若しくは情報又は損なうおそれのある情報であって研究の継続に影響を与えると考えられるものを知り得た場合（逸脱報告）

11.0　管理的事項

11.1　情報の取り扱い

11.1.1　情報の保管及び廃棄

情報の保存期間については、本研究中止又は終了後少なくとも 5 年間、あるいは研究結果発表後 3 年が経過した日までの間のどちらか遅い期日まで保存する。

11.1.2　情報を将来の研究に用いる可能性について（二次利用）

本研究では、研究目的で取得した研究対象者の情報の二次利用を行う可能性がある。なお、二次利用を行う場合には、改めて研究計画を立案し、倫理委員会における審査及び承認後、医学部長による実施の許可を得た上でなければ、利用することはできない。

11.2　研究資金・利益相反

11.2.1　研究資金等

本研究の研究資金は、以下のとおりとする。

- 近畿大学医学部　リハビリテーション医学　東本有司教授の配分研究費、水澤裕貴の科研費

11.2.2　利益相反の管理

本研究に関わるすべての研究者は、本学（又は所属する研究機関）の規定に従い、利益相反状況に関する自己申告を行う。当該申告に基づき、COI 委員会における審査・承認を受けた後でないと、研究を実施することはできない。また、当該申告に変更が生じた場合には、遅滞なく変更申請を行う。

11.3　情報公開

11.3.1　研究に関する情報公開

研究責任者は、本研究終了後、研究対象者の個人情報保護に関する措置を講じた上で、医学雑誌や学会において本研究の結果を公表する。本研究について、大学病院医療情報ネットワーク研究センター 臨床試験登録システム(UMIN-CRT)に臨床研究の登録を行い ID を取得する。

11.3.2　知的財産の帰属

本研究にかかる特許権等の経済的利益が生じた場合、その権利は本学に属し、研究対象者には属さない。

11.3.3　研究により得られた結果等の取扱い

本研究において実施した検査・観察項目により、研究対象者の医療上の問題が偶発的に発見される場合が想定される。その場合には、研究対象者に対してその旨を十分に説明するとともに、必要に応じて偶発的に発見された医療上の問題点に関する専門医への相談・紹介等を行う。

11.4　モニタリング及び監査

本研究は、侵襲を伴う研究には該当せず、モニタリング及び監査を実施しない。

12.0　実施体制

12.1　研究代表機関（研究事務局）

近畿大学医学部　リハビリテーション医学教室
近畿大学医学部　外科学教室

12.2　研究責任者

東本　有司　近畿大学医学部　リハビリテーション医学教室　臨床教授

12.3　研究分担者

水澤　裕貴　近畿大学病院　リハビリテーション部　理学療法士
野口　雅矢　近畿大学病院　リハビリテーション部　理学療法士
田村　友美　近畿大学病院　リハビリテーション部　言語聴覚士
白石　匡　　近畿大学病院　リハビリテーション部　主任　理学療法士

白石　治　　近畿大学医学部　外科学教室　准教授
平木　洋子　近畿大学医学部　外科学教室　医学部講師
加藤　寛章　近畿大学医学部　外科学教室　講師
安田　卓司　近畿大学医学部　外科学教室　主任教授

12.4　本研究に関する連絡先

水澤　裕貴　近畿大学病院　リハビリテーション部　理学療法士
内線番号（PHS）：8644
E メール：hiroki-mizusawa@med.kindai.ac.jp

13.0　参考文献

1. Pennathur A, Gibson MK, Jobe BA, Luketich JD. Oesophageal carcinoma. Lancet. 2013;381(9864):400–12.
2. Low, D. E., Kuppusamy, M. K., Alderson, D., Cecconello, I., Chang, A. C., Darling, G., ... & Wijnhoven, B. P. L. (2019). Benchmarking complications associated with esophagectomy. Annals of surgery, 269(2), 291-298.
3. Kawata, S., Hiramatsu, Y., Honke, J., Murakami, T., Booka, E., Matsumoto, T., ... & Takeuchi, H. (2024). Preoperative geniohyoid muscle mass in esophageal cancer patients is associated with swallowing function after esophagectomy. *Annals of Gastroenterological Surgery*, *8*(6), 1026-1035.
4. Vergara, J., Andreollo, N. A., Starmer, H. M., Miles, A., Baraçal-Prado, A. C. C., Junqueira, A. A., & Tincani, A. J. (2024). Swallowing Safety after Remote sub-total Esophagectomy: How Important is Tongue Pressure?. *Dysphagia*, 1-12.
5. Kojima, K., Fukushima, T., Kurita, D., Matsuoka, A., Ishiyama, K., Oguma, J., & Daiko, H. (2023). Perioperative decrease in tongue pressure is an intervenable predictor of aspiration after esophagectomy. *Dysphagia*, *38*(4), 1147-1155.
6. Zhang, W., Pan, H., Zong, Y., Wang, J., & Xie, Q. (2022). Respiratory muscle training reduces respiratory complications and improves swallowing function after stroke: a systematic review and Meta-analysis. *Archives of Physical Medicine and Rehabilitation, 103*(6), 1179-1191.
7. Hedström, J., Tuomi, L., Finizia, C., & Olsson, C. (2018). Correlations between patient-reported dysphagia screening and penetration–aspiration scores in head and neck cancer patients post-oncological treatment. Dysphagia, 33, 206-215.
8. Sugiya, R., Higashimoto, Y., Shiraishi, M., Tamura, T., Kimura, T., Chiba, Y.,... & Tohda, Y. (2022). Decreased tongue strength is related to skeletal muscle mass in COPD patients. Dysphagia, 37(3), 636-643.
9. 矢内敬子, 伊藤純平, 中平光彦, 榎木祐一郎, & 菅澤正. (2022). 唾液嚥下時の舌骨移動距離測定における超音波診断装置の有用性; 健常ボランティアでの検討. 頭頸部癌, 48(4), 351-355.
10. Macrae, P. R., Doeltgen, S. H., Jones, R. D., & Huckabee, M. L. (2012). Intra‐and inter‐rater reliability

for analysis of hyoid displacement measured with sonography. *Journal of clinical ultrasound*, *40*(2), 74-78.

11. Ogawa, N., Ohno, T., Kunieda, K., Watanabe, M., & Fujishima, I. (2024). A novel exercise to improve suprahyoid muscle area and intensity as evaluated by ultrasonography. Dysphagia, 1-9.
